# Moderating effect of work fatigue on the association between resilience and posttraumatic stress symptoms: a cross-sectional multi-country study among pharmacists during the COVID-19 pandemic

**DOI:** 10.1186/s13030-024-00300-0

**Published:** 2024-02-19

**Authors:** Samar Younes, Souheil Hallit, Irfan Mohammed, Sarah El Khatib, Anna Brytek-Matera, Shadrach Chinecherem Eze, Kenneth Egwu, Rawshan Jabeen, Nebojša Pavlović, Pascale Salameh, Michelle Cherfane, Marwan Akel, Chadia Haddad, Randa Choueiry, Feten Fekih-Romdhane, Katia Iskandar

**Affiliations:** 1 Department of Pharmaceutical Sciences, Faculty of Pharmacy, Lebanese International University, Beirut, Lebanon; 2 Institut National de Santé Publique, d’Épidémiologie Clinique et de Toxicologie-Liban (INSPECT- LB), Beirut, Lebanon; 3 School of Medicine and Medical Sciences, Holy Spirit University of Kaslik, P.O. Box 446, Jounieh, Lebanon; 4 Applied Science Research Center, Applied Science Private University, Amman, Jordan; 5 Department of Restorative Dentistry, School of Dentistry, Federal University of Pelotas, Pelotas, Brazil; 6 Department of Health Sciences, Faculty of Public Health, Lebanese University, Tripoli, Lebanon; 7 Eating Behavior Laboratory (EAT Younes et al. BioPsychoSocial Medicine, Institute of Psychology, University of Wroclaw, Wroclaw, Poland; 8 Federal Medical Centre, Makurdi Benue State, Makurdi, Nigeria; 9 Faculty of Pharmaceutical Sciences, University of Nigeria, Nsukka, Nigeria; 10 Department of Research & Development, Children’s Hospital Karachi, Karachi, Pakistan; 11 Department of Pharmacy, Faculty of Medicine, University of Novi Sad, Hajduk Veljkova 3, Novi Sad, 21000 Serbia; 12 Gilbert and Rose Marie Chagoury School of Medicine, Lebanese American University, Byblos, Lebanon; 13 Department of Primary Care and Population Health, University of Nicosia Medical School, Nicosia, Cyprus; 14 Faculty of Pharmacy, Lebanese University, Hadat, Lebanon; 15 Research Department, Psychiatric Hospital of the Cross, Jal Eddib, Lebanon; 16 School of Health Sciences, Modern University for Business and Science, Beirut, Lebanon; 17 Department of Medicinal Sciences, Faculty of Medicine, Lebanese University, Beirut, Lebanon; 18 Department of Psychiatry “Ibn Omrane”, Razi Hospital, Manouba, 2010 Tunisia; 19 Faculty of Medicine of Tunis, Tunis El Manar University, Tunis, Tunisia; 20 Institut supérieur de santé publique de l’université Saint-Joseph de Beyrouth, campus des sciences médicales, rue de Damas, BP 11-5076, Riad El Solh, Beyrouth, 1107 2180 Lebanon; 21 Faculty of Public Health-Section 2 (CERIPH), Lebanese University, Fanar, 90656 Lebanon

**Keywords:** Work fatigue, Resilience, Posttraumatic stress disorder, Pharmacists, COVID-19, Multi-country study

## Abstract

**Introduction:**

In the context of the COVID-19 pandemic, pharmacists, despite their vital contributions, have faced significant challenges that have impacted their mental well-being, potentially leading to the development of Post-Traumatic Stress symptoms (PTSS). The aim of this study was to investigate the role of work-related fatigue as a potential moderator in the relationship between pharmacists’ resilience and their likelihood of experiencing PTSS during the COVID-19 pandemic.

**Methods:**

A cross-sectional survey was conducted online in eight countries from January to December 2021, including Brazil, Lebanon, Nigeria, Pakistan, Poland, Serbia, and Tunisia. The mediation analysis was conducted using PROCESS MACRO (an SPSS add-on) v3.4 model 1, taking work fatigue as a moderator in the association between resilience and PTSS.

**Results:**

A total of 442 pharmacists were enrolled in this study (mean age = 33.91 ± 10.36 years) with 59.5% of them being females. The results were adjusted over country, gender, working in contact with COVID-19, working patients, working mandatory hours, working voluntary hours, age, household crowding index and number of months engaged in COVID-19. The interactions resilience by physical (Beta = 0.02; *p* = .029), mental (Beta = 0.02; *p* = .040) and emotional (Beta = 0.03; *p* = .008) work fatigue were significantly associated with PTSS; for pharmacists with low to moderate levels of physical (Beta = − 0.33; *p* < .001 and Beta = − 0.21; *p* = .001), mental (Beta = − 0.29; *p* < .001 and Beta = − 0.18; *p* = .006) and emotional (Beta = − 0.31; *p* < .001 and Beta = − 0.17; *p* = .008) work fatigue, higher resilience was significantly related to lower PTSS levels. However, for pharmacists with high levels of physical/mental/emotional work fatigue, the association between resilience and PTSS became non-significant.

**Conclusion:**

This study highlights the complex relationship between work-related fatigue, resilience, and PTSS in pharmacists. It emphasizes the need to address work-related fatigue for pharmacists’ psychological well-being during crises, offering insights for tailored support and interventions.

**Supplementary Information:**

The online version contains supplementary material available at 10.1186/s13030-024-00300-0.

## Introduction

Pharmacists are among the healthcare professionals who are the most accessible to patients. During the COVID-19 pandemic, they worked diligently to provide critical services that have shown to enhance patient outcomes and reduce healthcare costs [1–4]. Community pharmacists helped to reduce the spread of the coronavirus by raising patient awareness, especially by advising the patients on safety precautions and disseminating the necessary information [5]. They performed COVID-19 screening, dispensed the appropriate medications, engaged in home medication delivery and worked closely with other healthcare professionals and governmental agencies [6]. They were also occasionally the source of protective products like alcohol-based hand rub solutions and surgical masks [5]. Regarding hospital pharmacists, they directly collaborated and supported doctors, nurses, and other staff members by actively taking part in patient rounds, participating in the management of COVID-19 patients’ life-saving drugs, and implementing infectious disease control measures [1, 4, 6]. Throughout COVID-19, pharmacists encountered difficulties that affected all other health care workers (HCWs). Pharmacists in both community and hospital settings lacked enough personal protection equipment for facing the virus. They worked for long hours and had more responsibility and pressure while managing medication shortage and handling the growing number of anxious and frustrated patients. They were also at a higher risk of infection as a result of their work-related exposure leading to feelings of loneliness and isolation [7]. Therefore, pharmacists’ mental health and well-being were negatively impacted by these tasks because they made them feel more stressed, burdened, and frustrated.

Therefore, the psychological trauma endured by pharmacists during the COVID-19 pandemic can result in the development of Post-Traumatic Stress symptoms (PTSS) [8, 9]. Previously conducted studies have shown that prior infectious outbreaks like severe acute respiratory syndrome (SARS), the 2009 novel influenza A (H1N1), and Middle East respiratory syndrome (MERS) have resulted in higher rates of PTSS among HCWs [10]. Also, recent systematic reviews emphasized elevated levels of PTSS among HCWs during the COVID-19 pandemic [10–14]. Additionally, recent studies revealed a general decline in the mental health of pharmacists [15] and indicated that pharmacists experienced rates of burnout and secondary traumatic stress (STS) similar to other HCWs. Approximately, 47% of pharmacists have reported experiencing burnout syndrome, with 51% of that burnout attributed to the impact of the COVID-19 pandemic [16]. Hence, the elevated risk of PTSS among HCWs, including pharmacists has been well-documented, and understanding the factors that mitigate or exacerbate this risk is of paramount importance [17].

Resilience, the ability to adapt and bounce back from adversity, has emerged as a key psychological resource that may play a significant role in potentially reducing the negative effects of stresses, fostering psychological wellbeing, and protecting against the development of PTSS among HCWs [18–20]. Amidst the turmoil, pharmacists have shown remarkable resilience, yet they have not been immune to the psychological toll of the pandemic [21]. As a protective factor, resilience empowers individuals to better navigate difficulties and traumatic experiences, fostering superior emotional regulation, problem-solving skills, and a sense of control that can diminish their vulnerability to PTSS [22]. Among those already affected, resilient individuals often exhibit milder symptoms, faster recovery, and an aptitude for utilizing adaptive coping strategies, which collectively ameliorate PTSS’s impact [23, 24]. While there is limited specific evidence among pharmacists who have been frontline workers during the pandemic, it is reasonable to assume that those with higher levels of resilience may be better equipped to handle the challenges they face in their work such as increased workload, stress, and potential trauma associated with their roles [25]. Resilience may empower pharmacists to confront these challenges more effectively, engage in self-care practices, and seek necessary support, thereby reducing the risk of PTSS or secondary traumatic stress [26, 27]. However, it’s important to acknowledge that resilience is not an absolute shield against PTSS, as its development is influenced by multiple factors, including the nature and severity of traumatic events [24]. The extent to which resilience lowers the chance of acquiring PTSS in pharmacists is still an understudied aspect of their pandemic experience.

Studies have shown reduced resilience levels among individuals who experience prolonged periods of work fatigue. Work fatigue is defined as a condition that has significant implications for employees’ health, work performance, attitude, and safety [28, 29]. It encompasses extreme exhaustion and tiredness experienced during and after workdays. Work fatigue can be categorized into three main types: physical, emotional, and mental. Physical work fatigue involves extreme physical tiredness and diminished physical capabilities. Emotional fatigue is characterized by emotional exhaustion and reduced capacity for emotional engagement during or after work. Mental work fatigue pertains to cognitive tiredness that hinders engagement in cognitive tasks during or after work [28, 30]. Research conducted in various occupational settings, including pharmacists, has demonstrated that chronic workplace stress, excessive workload, and long working hours can reduce an individual’s resilience [31–35]. This weakened resilience can manifest as increased vulnerability to stressors, impaired emotional regulation, and decreased problem-solving abilities [36, 37]. On the other hand, there is evidence of a strong relationship between fatigue and the development of PTSS. Individuals who experience prolonged or severe fatigue, especially in the context of traumatic events such as the COVID-19 pandemic, are at an increased risk of developing PTSS [38]. This is evident in studies conducted on various populations, including military personnel, first responders, and healthcare workers, where those exposed to extreme fatigue due to long working hours, high stress, and overwhelming demands have shown a higher likelihood of developing PTSS [39–41].

It is therefore reasonable to hypothesize that work fatigue may interact with an individual’s resilience, influencing its protective effect against PTSS. Specifically, It can be suggested that the presence of significant work fatigue may reduce the protective role of resilience, potentially making individuals more vulnerable to PTSS. In contrast, when work fatigue is minimal, resilience may have a more potent protective effect [42]. However, this complex interaction between resilience, work fatigue, and PTSS among pharmacists remains an area that needs plenty of research [43, 44].

Resilience has been shown to be protective against adverse mental health outcomes when stress is present [45], unless their effects become weakened by moderating factors, such as work fatigue. Investigating possible moderators in the relationship between resilience and PTSS may pave the way for targeted interventions and developing support strategies that empower pharmacists to navigate the ongoing challenges of the pandemic while safeguarding their psychological well-being [46]. Therefore, the current study was conducted to investigate the role of work-related fatigue as a potential moderator in the relationship between pharmacists’ resilience and their likelihood of experiencing PTSS during the COVID-19 pandemic. Specifically, the study aimed to explore whether the protective effect of resilience against PTSS is influenced or moderated by the levels of work-related fatigue experienced by pharmacists in multiple countries during this global health crisis.

## Methods

Study design and sampling.

A cross-sectional survey was conducted online in eight countries from January to December 2021, including Brazil, Lebanon, Nigeria, Pakistan, Poland, Serbia, and Tunisia. The choice of a multi-country design was made to provide a broader overview of the relation between study variables in various countries and regions of the world. The long period of data collection was due to the difficulty in availability of collaborators from the different countries involved, and in collecting data from pharmacists in the context of the COVID-19 situation, as this time of crisis substantially affected the daily practice of this population and required their priorities to be modified. This article represents an ongoing multinational collaboration on relevant public health topics between researchers from these countries. The questionnaire was created using Google Forms, a cloud-based survey tool powered by Google™ and the questionnaire link was distributed online, using snowball sampling to enroll participants [47]. A convenient sample of at least 100 participants per country was set due to difficulties collecting data during the pandemic. Our samples consisted of pharmacists working in hospital or community pharmacy settings from eight countries. An introductory paragraph was included at the beginning of the link explaining the objectives of the study, while assuring participants about anonymity and confidentiality of their responses. After providing digital informed consent, participants were asked to complete a questionnaire. Participants completed the survey voluntarily and without remuneration.

Questionnaire.

The online survey was formulated in English and translated to Arabic, Portuguese, Serbian and Polish according to the World Health Organization (WHO) translation guidelines. The research teams collaborated with a certified translator, consulted to detect discrepancies, solve them, validate and translate the questionnaire into the local language; this version was back-translated to English by a second certified translator in each country. A pilot test was done on 20 participants to make sure that the questions were well understood.

Sample size calculation.

To calculate the sample size, we used G*Power software. The minimum required sample size was 389 participants, considering an alpha error of 5%, a power of 80%, a minimal model r-square of 5%, and allowing 15 predictors for inclusion in the model.

Data collection.

The first section of the questionnaire assessed the demographic characteristics, including age, gender, marital status, country of origin, primary work setting, the household crowding index, and financial distress or well-being scale.

The household crowding index is determined as follows: dividing the number of people by the number of rooms in the house except for the bathrooms and kitchen [48]. The InCharge Financial Distress/Financial Well-Being Scale is a set of 8 self-reported items representing the perceived financial well-being or distress status. Each response represents the participant financial state reflected on a continuum ranging from 1 (financial distress) to 10 (financial well-being) [49]. In this study, the Cronbach alpha value was 0.842.

The second section examined work exposure to COVID-19 and institutional support and demand during the pandemic. The third part intended to determine the sources of fear and factors contributing to resilience.

Outcomes measures.

Four scales were used to assess the psychological impact of the COVID-19 pandemic on healthcare professionals.

1. Three-Dimensional Work Fatigue Inventory (3D-WFI).

The Three-Dimensional Work Fatigue Inventory (3D-WFI) is a validated measure for the evaluation of work fatigue [28, 29]. According to Frone & Tidwel, work fatigue is a status of extreme tiredness and declined functional capacity to engage in physical, cognitive, and emotional activity experienced during and at the end of a work day [28]. The 3D-WFI consists of 18 items divided into three commensurate domains containing every six items, with three representing extreme tiredness and three others assessing reduced functional capacity. Respondents were provided a 5-point Likert scale to describe work fatigue during the past 12 months based on the three energetic resources (physical, mental and emotional). The scale ranged from never (score 0), less than once per month (score 1), at least once per month (score 2), at least once a week (score 3), and every day (score 4). Higher scores indicate higher work fatigue. In this study, Cronbach’s alpha was 0.866, 0.887, and 0.905 for the physical, mental, and emotional subscales).

2. The Impact of Event scale (IES-6).

The Impact of Event scale (IES-6) [50] is an abbreviated version of the IES-R [51]. The IES-6 scales were widely used in the literature to assess the occurrence of PTSS due to COVID-19 [52–57]. The IES-6 scale consists of 6-items that represent three subscales describing the PTSS, such as avoidance (“I was aware that I still had a lot of feelings about it, but I didn’t deal with them” and “I tried not to think about it”), intrusion (“Other things kept making me think about it” and “I thought about it when I did not mean to”), and hyperarousal (“I felt watchful or on guard” and “I had trouble concentrating”). Each subscale includes two items. The respondents were asked to indicate on a 5-point Likert scale their feelings during the COVID-19 pandemic before they got vaccinated. The Likert scale ranged from never (score 0), seldom (score 1), sometimes (score 2), often (score 3), and almost always (score 4). The summed score ranged from 0 to 24. Higher scores indicate higher PTSS symptoms. In this study, Cronbach’s Alpha is 0.830.

3. Brief Resilience Scale (BRS).

The BRS is a tool for determining one ability to recover, cope, and bounce back after facing stressful events [58]. The BRS is a validated measure of resilience with a demonstrated Cronbach’s alpha ranging from 0.80 to 0.91 and a one-month test-retest reliability (ICC) of 0.69 [58]. It consists of six items, three positively and three negatively worded items. Respondents were provided a 5-point Likert scale to assess their resilience in facing the COVID-19 pandemic hurdles. The scale ranged from Strongly disagree (score 1) to strongly agree (score 5). The negative items (“I have a hard time making it through stressful events “, “It is hard for me to snap back when something bad happens” and “I tend to take a long time to get over set-backs in my life”) were scored by reverse coding. The total average score ranged from 1.00 to 5.00, where a value of 3.0–4.3 is considered a good level of resilience [59]. In this study, Cronbach’s Alpha is 0.500).

### Statistical analysis

The SPSS software v.26 was used for the statistical analysis. The IES score was considered normally distributed since the skewness (= 0.217) and kurtosis ( = − 0.228) values varied between ± 1. The Student *t* test was used to compare two means and the Pearson test to correlate two continuous variables. The moderation analysis was conducted using PROCESS MACRO (an SPSS add-on) v3.4 model 1, taking work fatigue as a moderator in the association between resilience and PTSS. Interaction terms were probed by examining the association of the predictor with PTSS at the mean, 1 SD below the mean and 1 SD above the mean of the moderators (physical/mental/emotional work fatigue). Results were adjusted over all variables that showed a *p* < .25 in the bivariate analysis. *P* < .05 was deemed statistically significant.

## Results

A total of 442 pharmacists were enrolled in this study (mean age = 33.91 ± 10.36 years) with 59.5% of them being females. Other characteristics of the sample are shown in Table 1.

**Table 1:** Sociodemographic and other characteristics of the participants (*n* = 442)

Variable	n (%)
Country	
Developed	46 (10.4%)
Developing	396 (89.6%)
**Gender**	
Male	179 (40.5%)
Female	263 (59.5%)
**Marital status**	
Single / divorced	285 (64.5%)
Married	157 (35.5%)
**Specialty**	
Hospital pharmacy	206 (46.6%)
Community pharmacy	236 (53.4%)
**Working in contact with COVID-19 patients**	
No	215 (48.6%)
Yes	227 (51.4%)
**Working voluntarily hours**	
No	158 (35.7%)
Yes	284 (64.3%)
**Working mandatory hours**	
No	301 (68.1%)
Yes	141 (31.9%)
	**Mean ± SD**
PTSS (IES scores)	8.17 ± 4.82
Resilience	18.28 ± 3.78
Physical work fatigue	16.61 ± 4.99
Mental work fatigue	16.07 ± 5.12
Emotional work fatigue	15.32 ± 5.37
Age (years)	33.91 ± 10.36
Household crowding index (person/room)	0.85 ± 0.80
Financial satisfaction	37.10 ± 1257
Number of months engaged in COVID	7.55 ± 5.86
Additional hours	8.06 ± 13.95

### Bivariate analysis

The bivariate analysis results are shown in Tables 2 and 3. A higher IES mean score was significantly found in females compared to males, and in participants who did not work mandatory hours. Furthermore, higher resilience was significantly associated with lower PTSS scores, whereas higher mental work fatigue was significantly associated with higher PTSS scores.

**Table 2:** Bivariate analysis of the categorical variables associated with PTSS

Variable	Mean ± SD	t	df	p
**Country**		-1.199	440	0.231
Developed	8.08 ± 4.74			
Developing	8.98 ± 5.45			
**Gender**		-2.564	440	**0.011**
Male	7.46 ± 4.49			
Female	8.65 ± 4.99			
**Marital status**		0.001	440	1
Single / divorced	8.17 ± 4.96			
Married	8.17 ± 4.58			
**Specialty**		0.624	440	0.533
Hospital pharmacy	8.33 ± 4.67			
Community pharmacy	8.04 ± 4.95			
**Working in contact with COVID-19 patients**		-1.357	440	0.176
No	7.85 ± 5.20			
Yes	8.48 ± 4.42			
**Working voluntarily hours**		-1.326	440	0.186
No	7.73 ± 5.59			
Yes	8.42 ± 4.33			
**Working mandatory hours**		2.213	440	**0.028**
No	8.54 ± 4.49			
Yes	7.38 ± 5.40			

Numbers in bold indicate significant *p* values.

**Table 3:** Correlation matrix of continuous variables

Variable	1	2	3	4	5	6	7	8	9	10
1. PTSS	1									
2. Resilience	− 0.19***	1								
3. Physical work fatigue	− 0.04	0.02	1							
4. Mental work fatigue	0.12**	− 0.11*	0.71***	1						
5. Emotional work fatigue	0.06	− 0.16**	0.63***	0.78***	1					
6. Age	0.09	0.001	− 0.13**	− 0.13**	− 0.07	1				
7. Household crowding index	− 0.09	0.09	0.12*	0.12**	0.08	− 0.11*	1			
8. Financial satisfaction	0.001	0.03	− 0.31***	− 0.26***	− 0.33***	0.18***	− 0.06	1		
9. Number of months engaged in COVID	0.09	0.15**	0.10*	0.08	0.02	0.17***	− 0.05	0.01	1	
10. Additional hours	0.06	− 0.03	0.12*	0.13**	0.06	0.06	− 0.04	− 0.12*	0.14**	1

Numbers in the table reflect Pearson correlation coefficients; **p < .01; ***p < .001.

### Moderation analysis with psychological distress taken as the dependent variable

The details of the moderation analysis of work fatigue taken as a moderator in the association between resilience and PTSS, are summarized in Table 4. The results were adjusted over country, gender, working in contact with COVID-19, working patients, working mandatory hours, working voluntary hours, age, household crowding index and number of months engaged in COVID-19. The interactions resilience by physical (Beta = 0.02; *p* = .029), mental (Beta = 0.02; *p* = .040) and emotional (Beta = 0.03; *p* = .008) work fatigue were significantly associated with PTSS (Table 4); for pharmacists with low to moderate levels of physical (Beta = − 0.33; *p* < .001 and Beta = − 0.21; *p* = .001), mental (Beta = − 0.29; *p* < .001 and Beta = − 0.18; *p* = .006) and emotional (Beta = − 0.31; *p* < .001 and Beta = − 0.17; *p* = .008) work fatigue, higher resilience was significantly related to lower PTSS levels. For pharmacists with high levels of physical/mental/emotional work fatigue, the association between resilience and PTSS became non-significant (Table 5).

**Table 4:** Moderation analysis taking resilience as the independent variable, work fatigue subscales as moderators and PTSS scores as the dependent variable

Model 1: Physical work fatigue as the moderator.				
	Beta	t	P	95% CI
Resilience	− 0.61	-3.263	**0.001**	− 0.98; − 0.24
Physical work fatigue	− 0.49	-2.254	**0.025**	− 0.92; − 0.06
Interaction resilience by physical work fatigue	0.02	2.196	**0.029**	0.003; 0.046*
**Model 2: Mental work fatigue as the moderator.**				
Resilience	− 0.52	-3.125	**0.002**	− 0.84; − 0.19
Mental work fatigue	− 0.30	-1.434	0.152	− 0.70; 0.11
Interaction resilience by mental work fatigue	0.02	2.058	**0.040**	0.001; 0.041*
**Model 3: Emotional work fatigue as the moderator.**				
Resilience	− 0.57	-3.836	**< 0.001**	− 0.86; − 0.28
Emotional work fatigue	− 0.46	-2.377	**0.018**	− 0.84; − 0.08
Interaction resilience by emotional work fatigue	0.03	2.649	**0.008**	0.01; 0.05*

*indicates significant moderation; numbers in bold indicate significant *p* values.

**Table 5:** Conditional effects of the focal predictor (resilience) at values of the moderator (work fatigue)

	Beta	t	p	95% CI
Model 1: Physical work fatigue as the moderator.				
Low (= 11.61)	− 0.33	-4.17	**< 0.001**	− 0.48; − 0.17
Moderate (= 16.59)	− 0.21	-3.24	**0.001**	− 0.33; − 0.08
High (= 21.58)	− 0.08	− 0.93	0.354	− 0.26; 0.09
**Model 2: Mental work fatigue as the moderator.**				
Low (= 10.95)	− 0.29	-3.81	**< 0.001**	− 0.43; − 0.14
Moderate (= 16.08)	− 0.18	-2.79	**0.006**	− 0.30; − 0.05
High (= 21.20)	− 0.07	− 0.79	0.432	− 0.25; 0.11
**Model 3: Emotional work fatigue as the moderator.**				
Low (= 9.93)	− 0.31	-4.24	**< 0.001**	− 0.46; − 0.17
Moderate (= 15.31)	− 0.17	-2.65	**0.008**	− 0.30; − 0.04
High (= 20.69)	− 0.03	− 0.36	0.716	− 0.22; 0.15

Numbers in bold indicate significant *p* values.

## Discussion

The COVID-19 pandemic has posed unprecedented challenges to healthcare professionals across the globe, and pharmacists, as essential frontline workers, have been no exception. The multifaceted demands placed on pharmacists during this crisis have been accompanied by high prevalence rates of PTSS [60, 61] and low levels of resilience [25, 62]. Against this backdrop, this cross-sectional multi-country study was conducted to delve into the intricate interplay between resilience and PTSS among pharmacists during the COVID-19 pandemic. Specifically, it was investigated how varying levels of work fatigue, stemming from the physical, mental, and emotional toll of their profession, may moderate the relationship between resilience and PTSS. Our results showed that in pharmacists experiencing mild to moderate physical, mental, and emotional work fatigue, greater resilience was strongly linked to reduced levels of PTSS. However, for pharmacists facing high levels of physical, mental, or emotional work fatigue, the connection between resilience and PTSS did not show statistical significance. These findings not only address a critical gap in our understanding of pharmacist well-being during a global health crisis, but also offer insights that can inform targeted interventions aimed at bolstering the mental health and resilience of these vital healthcare professionals.

One of the findings of this study pertain to pharmacists’ PTSS levels in relation to resilience. A robust and significant association was found between higher levels of resilience and lower PTSS scores, suggesting that pharmacists exhibiting higher levels of resilience were less likely to report PTSS. This finding is expected, on the basis of the well-established protective role of resilience in buffering against the psychological toll of high-stress situations [63–66]. Pharmacists who displayed greater resilience exhibited a remarkable ability to cope with the demands and uncertainties of their profession during this crisis, resulting in reduced PTSS. In a prior investigation carried out in Lebanon, it was found that community pharmacists exhibited a relatively low level of resilience during the COVID-19 pandemic, and this was associated with elevated rates of burnout [25]. Another study conducted also on Qatari pharmacists during the pandemic revealed that they encountered a moderate level of burnout, yet they exhibited a moderate level of resilience, suggesting their capacity to effectively cope with challenges [62].

Additionally, our findings also unveiled a compelling relationship between higher mental work fatigue and elevated PTSS scores among pharmacists. This result implies that pharmacists who experienced heightened mental fatigue, possibly stemming from the complexity and volume of their tasks, such as navigating complex medication interactions or managing a high number of prescriptions, took on the mental well-being of these healthcare professionals. The mental fatigue associated with their professional responsibilities during the pandemic seemed to amplify the impact of stressors on their psychological well-being. Interviews with pharmacists in Wisconsin showed that they grappled with mental exhaustion, particularly on days marked by heavier workloads. This mental fatigue had an impact on their interactions with both fellow staff members, patients, and family members [67]. Additionally, another study showed that most pharmacists were identified with moderate-high likelihood of burnout and moderate-high probability of secondary traumatic stress at rates which are significantly higher compared with rates early in the pandemic [68].

Moreover, a moderation analysis was conducted in this study to examine the moderating role of work-related fatigue on the relationship between resilience and PTSS. This approach provides valuable insights into the complex interplay between personal resilience, work-related fatigue, and post-traumatic stress outcomes, shedding light on potential factors that could mitigate or exacerbate the effects of resilience on PTSS in occupational settings.

Our analysis demonstrated a significant correlation between increased resilience and reduced PTSS levels among pharmacists who experienced low to moderate levels of physical, mental, and emotional fatigue in their work. However, the association between resilience and PTSS became non-significant for pharmacists with high levels of the three work fatigue dimensions. In essence, when pharmacists exhibit greater resilience and operate within the bounds of manageable work-related fatigue, they tend to report lower levels of PTSS. This suggests that resilience may function as a protective mechanism, mitigating the impact of work-related challenges on their mental health, especially when the fatigue associated with their professional duties remains within reasonable limits. On the other hand, when pharmacists experienced high levels of physical and mental fatigue due to their job demands, the buffering effect of resilience on PTSS appears to diminish or weaken. In such cases, the impact of work fatigue may overwhelm the potential protective influence of resilience. An abundance of research has focused on identifying factors affecting mental health in pharmacists [7, 15, 61], however there is a paucity of research examining the moderating effect of work fatigue on the association between resilience and PTSS among them. Therefore, it was not feasible to compare our study results with similar research because there was a scarcity of existing studies on the same topic. Nevertheless, numerous studies link resilience with burnout, fatigue and stress in HCWs [69–72]. In a prior longitudinal study, it was determined that resilience had a mitigating effect on nurse fatigue [73]. Another study involving healthcare professionals found that individuals with higher levels of resilience reported improved perceived immune function and a reduction in both physical and psychological symptom [74]. Also, multiple studies conducted to explore the relationship between exhaustion and PTSS resulting from excessive workplace stress have consistently demonstrated a robust and positive correlation between burnout and PTSS [75–77].

### Clinical implications

The current study carries significant implications for both the pharmacy profession and the broader healthcare sector. The findings shed light on the crucial role of resilience in mitigating the psychological impact of a global health crisis on healthcare workers. Recognizing the role of resilience in buffering the impact of work-related stressors on mental health and reducing the development of PTSS highlights the need for resilience-building interventions and support systems within the pharmaceutical field to enhance pharmacists’ mental well-being during such crises. Furthermore, the identification of work fatigue as a moderator underscores the importance of managing fatigue and workload through implementing fatigue management strategies, optimizing work hours, and prioritizing the well-being of these professionals to help safeguard their resilience and reduce the risk of developing PTSS. These insights can inform policy decisions and workplace strategies to better protect the mental health of pharmacists and other frontline HCWs during and beyond pandemics, ultimately ensuring their ability to provide quality care to patients.

### Strengths and limitations

The present study possesses several notable strengths and limitations. A key strength lies in its multi-country approach, which enhances the generalizability of findings offering a broad perspective on the relationship between resilience, work fatigue, and PTSS among pharmacists during the COVID-19 pandemic. Furthermore, its timeliness and relevance to current events provide valuable insights into the unique challenges faced by pharmacists and healthcare professionals during a critical period. The utilization of resilience as a variable and the study’s quantitative approach strengthens the empirical foundation of the study. However, several limitations should be acknowledged. The cross-sectional design of the study restricts the ability to determine causality, and reliance on self-reported data introduces potential bias. Subjects were recruited from January to December 2021; therefore, participants’ conditions might have varied depending on the time of the survey. The quality of the survey may not be high in online surveys; in addition, we could not know which participants answered the survey in an inappropriate way to exclude them. Additionally, the generalizability of findings to other professions may be limited, and the study’s focus on a specific set of variables leaves other potential contributors to pharmacists’ mental well-being unexplored. Despite these limitations, the study significantly contributes to our understanding of the mental health dynamics faced by pharmacists in high-stress contexts like the COVID-19 pandemic and underscores the need for future research to delve further into the complexities of this issue, especially during times of heightened stress and crisis.

## Conclusion

In summary, this study highlights the intricate interplay between resilience, work-related fatigue, and PTSS levels among pharmacists. It highlights the role of resilience in reducing PTSS and underscore the imperative of addressing mental work fatigue to safeguard the psychological health of pharmacists during challenging times. It offers a nuanced perspective on the factors influencing mental well-being among pharmacists during times of crisis, providing valuable insights for tailored support and interventions that not only foster resilience-building strategies but also address the unique challenges of work fatigue within the pharmacy profession.

### Supplementary Information

**Additional file 1.**

**Additional file 2.**

## Data Availability

All data generated or analyzed during this study are not publicly available due the restrictions from the ethics committee. Reasonable requests can be addressed to the corresponding author (SH).
