# Supplementary material for: Moderating effect of work fatigue on the association between resilience and posttraumatic stress symptoms: a cross-sectional multi-country study among pharmacists during the COVID-19 pandemic
Source: Biopsychosoc Med. 2024 Feb 19;18:4. doi: 10.1186/s13030-024-00300-0 (PMC10875825; doi:10.1186/s13030-024-00300-0)
Supplement: Supplementary file 1 — Additional file 1. [file 13030_2024_300_MOESM1_ESM.docx]

**Moderating effect of work fatigue on the association between resilience and Posttraumatic Stress symptoms: A cross-sectional multi-country study among pharmacists during the COVID-19 pandemic**

Samar Younes^* 1,2^, Souheil Hallit^3,4,5^, Irfan Mohammed^6^, Sarah El Khatib^2,7^, Anna Brytek-Matera^8^, Shadrach Chinecherem Eze^9^, Kenneth Egwu^10^, Rawshan Jabeen^11^, Nebojša Pavlović^12^, Pascale Salameh^2,13,14,15^, Michelle Cherfane^2,13^ ,Marwan Akel^2,16^, Chadia Haddad^2,5,13,17^, Randa Choueiry^18^, Feten Fekih-Romdhane^19,20^, Katia Iskandar^1,2,21^

1. School of Pharmacy, Lebanese International University, Lebanon
2. Institut National de Santé Publique, d’Épidémiologie Clinique et de Toxicologie-Liban (INSPECT-LB), Beirut, Lebanon
3. School of Medicine and Medical Sciences, Holy Spirit University of Kaslik, P.O. Box 446, Jounieh, Lebanon.
4. Applied Science Research Center, Applied Science Private University, Amman, Jordan
5. Research Department, Psychiatric Hospital of the Cross, Jal Eddib, Lebanon
6. Department of restorative dentistry, School of Dentistry, Federal University of Pelotas, Pelotas, Brazil.
7. Department of Health Sciences, Faculty of Public Health, Lebanese University, Tripoli, Lebanon.
8. Eating Behavior Laboratory (EAT Lab), Institute of Psychology, University of Wroclaw, Wroclaw, Poland.
9. Federal Medical Centre, Makurdi Benue State, Makurdi, Nigeria.
10. Faculty of Pharmaceutical Sciences, University of Nigeria, Nsukka, Nigeria.
11. Department of Research & development, Children's hospital Karachi, Karachi, Pakistan.
12. Department of Pharmacy, Faculty of Medicine, University of Novi Sad, Hajduk Veljkova 3, 21000 Novi Sad, Serbia.
13. Department of Medical Sciences, School of Medicine, Lebanese American University, Byblos, Lebanon.
14. Department of Primary Care and Population Health, University of Nicosia Medical School, Nicosia, Cyprus.
15. Faculty of Pharmacy, Lebanese University, Hadat, Lebanon
16. Department of Pharmaceutical sciences, Faculty of Pharmacy, Lebanese International University, Lebanon.
17. School of Health Sciences, Modern University for Business and Science, Beirut, Lebanon.
18. Department of Medicinal sciences, Faculty of Medicine, Lebanese University, Beirut, Lebanon
19. Department of psychiatry “Ibn Omrane”, Razi hospital, Manouba, 2010 Tunisia.
20. Faculty of Medicine of Tunis, Tunis El Manar University, Tunis, Tunisia
21. Department of Health sciences, Faculty of Public Health, Lebanese University, Beirut, Lebanon.

Feten Fekih-Romdhane and Katia Iskandar are last coauthors.

Corresponding author: Katia Iskandar. School of Pharmacy, Lebanese International University, Lebanon. Email: [katia_iskandar@hotmail.com](mailto:katia_iskandar@hotmail.com) and Souheil Hallit, School of Medicine and Medical Sciences, Holy Spirit University of Kaslik, P.O. Box 446, Jounieh, Lebanon. Email: [souheilhallit@hotmail.com](mailto:souheilhallit@hotmail.com)

Samar Younes: [samar.younes@liu.edu.lb](mailto:samar.younes@liu.edu.lb)

Souheil Hallit: [souheilhallit@hotmail.com](mailto:souheilhallit@hotmail.com)

Irfan Mohammed: [irfan_dentart@yahoo.com](mailto:irfan_dentart@yahoo.com)

Sarah El Khatib: [sarah.khatib@liu.edu.lb](mailto:sarah.khatib@liu.edu.lb)

Anna Brytek-Matera: [anna.brytek-matera@uwr.edu.pl](mailto:anna.brytek-matera@uwr.edu.pl)

Shadrach Chinecherem Eze: [shadrach1001@gmail.com](mailto:shadrach1001@gmail.com)

Kenneth Egwu: [kenegwukenegwu@gmail.com](mailto:kenegwukenegwu@gmail.com)

Rawshan Jabeen: [rawshanjabeen@gmail.com](mailto:rawshanjabeen@gmail.com)

Nebojša Pavlović: [nebojsa.pavlovic@mf.uns.ac.rs](mailto:nebojsa.pavlovic@mf.uns.ac.rs)

Pascale Salameh: [pascalesalameh1@hotmail.com](mailto:pascalesalameh1@hotmail.com)

Michelle Cherfane: [michellecherfane@gmail.com](mailto:michellecherfane@gmail.com)

Marwan Akel: [Marwan.akel@liu.edu.lb](mailto:Marwan.akel@liu.edu.lb)

Chadia Haddad: [chadia_9@hotmail.com](mailto:chadia_9@hotmail.com)

Randa Choueiry: [rchoueirim@hotmail.com](mailto:rchoueirim@hotmail.com)

Feten Fekih-Romdhane: [feten.fekih@gmail.com](mailto:feten.fekih@gmail.com)

Katia Iskandar: [Katia_iskandar@hotmail.com](mailto:Katia_iskandar@hotmail.com)

**Abstract:**

**Introduction:** In the context of the COVID-19 pandemic, pharmacists, despite their vital contributions, have faced significant challenges that have impacted their mental well-being, potentially leading to the development of Post-Traumatic Stress symptoms (PTSS). The aim of this study was to investigate the role of work-related fatigue as a potential moderator in the relationship between pharmacists' resilience and their likelihood of experiencing PTSS during the COVID-19 pandemic.

**Methods:** A cross-sectional survey was conducted online in eight countries from January to December 2021, including Brazil, Lebanon, Nigeria, Pakistan, Poland, Serbia, and Tunisia. The mediation analysis was conducted using PROCESS MACRO (an SPSS add-on) v3.4 model 1, taking work fatigue as a moderator in the association between resilience and PTSS.

**Results:** A total of 442 pharmacists were enrolled in this study (mean age= 33.91 ± 10.36 years) with 59.5% of them being females. The results were adjusted over country, gender, working in contact with COVID-19, working patients, working mandatory hours, working voluntary hours, age, household crowding index and number of months engaged in COVID-19. The interactions resilience by physical (Beta = .02; *p* = .029), mental (Beta = .02; *p* = .040) and emotional (Beta = .03; *p* = .008) work fatigue were significantly associated with PTSS; for pharmacists with low to moderate levels of physical (Beta = -.33; *p* < .001 and Beta = -.21; p = .001), mental (Beta = -.29; *p* < .001 and Beta = -.18; p = .006) and emotional (Beta = -.31; *p* < .001 and Beta = -.17; p = .008) work fatigue, higher resilience was significantly related to lower PTSS levels. However, for pharmacists with high levels of physical/mental/emotional work fatigue, the association between resilience and PTSS became non-significant.

**Conclusion:** This study highlights the complex relationship between work-related fatigue, resilience, and PTSS in pharmacists. It emphasizes the need to address work-related fatigue for pharmacists' psychological well-being during crises, offering insights for tailored support and interventions.

**Keywords:** Work fatigue; Resilience; Posttraumatic Stress Disorder; Pharmacists; COVID-19; Multi-country study.

**Intr****oduction**

Pharmacists are among the healthcare professionals who are the most accessible to patients. During the COVID-19 pandemic, they worked diligently to provide critical services that have shown to enhance patient outcomes and reduce healthcare costs [1–4]. Community pharmacists helped to reduce the spread of the coronavirus by raising patient awareness, especially by advising the patients on safety precautions and disseminating the necessary information [5]. They performed COVID-19 screening, dispensed the appropriate medications, engaged in home medication delivery and worked closely with other healthcare professionals and governmental agencies [6]. They were also occasionally the source of protective products like alcohol-based hand rub solutions and surgical masks [5]. Regarding hospital pharmacists, they directly collaborated and supported doctors, nurses, and other staff members by actively taking part in patient rounds, participating in the management of COVID-19 patients' life-saving drugs, and implementing infectious disease control measures [1,6,7]. Throughout COVID-19, pharmacists encountered difficulties that affected all other health care workers (HCWs). Pharmacists in both community and hospital settings lacked enough personal protection equipment for facing the virus. They worked for long hours and had more responsibility and pressure while managing medication shortage and handling the growing number of anxious and frustrated patients. They were also at a higher risk of infection as a result of their work-related exposure leading to feelings of loneliness and isolation [8]. Therefore, pharmacists' mental health and well-being were negatively impacted by these tasks because they made them feel more stressed, burdened, and frustrated.

Therefore, the psychological trauma endured by pharmacists during the COVID-19 pandemic can result in the development of Post-Traumatic Stress symptoms (PTSS) [9,10]. Previously conducted studies have shown that prior infectious outbreaks like severe acute respiratory syndrome (SARS), the 2009 novel influenza A (H1N1), and Middle East respiratory syndrome (MERS) have resulted in higher rates of PTSS among HCWs [12]. Also, recent systematic reviews emphasized elevated levels of PTSD among HCWs during the COVID-19 pandemic [12–16]. Additionally, recent studies revealed a general decline in the mental health of pharmacists [17] and indicated that pharmacists experienced rates of burnout and secondary traumatic stress (STS) similar to other HCWs. Approximately, 47% of pharmacists have reported experiencing burnout syndrome, with 51% of that burnout attributed to the impact of the COVID-19 pandemic [18]. Hence, the elevated risk of PTSD among HCWs, including pharmacists has been well-documented, and understanding the factors that mitigate or exacerbate this risk is of paramount importance [19].

Resilience, the ability to adapt and bounce back from adversity, has emerged as a key psychological resource that may play a significant role in potentially reducing the negative effects of stresses, fostering psychological wellbeing, and protecting against the development of PTSS among HCWs [20–22]. Amidst the turmoil, pharmacists have shown remarkable resilience, yet they have not been immune to the psychological toll of the pandemic [23]. As a protective factor, resilience empowers individuals to better navigate difficulties and traumatic experiences, fostering superior emotional regulation, problem-solving skills, and a sense of control that can diminish their vulnerability to PTSS [24]. Among those already affected, resilient individuals often exhibit milder symptoms, faster recovery, and an aptitude for utilizing adaptive coping strategies, which collectively ameliorate PTSS's impact [25,26]. While there is limited specific evidence among pharmacists who have been frontline workers during the pandemic, it is reasonable to assume that those with higher levels of resilience may be better equipped to handle the challenges they face in their work such as increased workload, stress, and potential trauma associated with their roles [27]. Resilience may empower pharmacists to confront these challenges more effectively, engage in self-care practices, and seek necessary support, thereby reducing the risk of PTSS or secondary traumatic stress [28,29]. However, it's important to acknowledge that resilience is not an absolute shield against PTSS, as its development is influenced by multiple factors, including the nature and severity of traumatic events [26]. The extent to which resilience lowers the chance of acquiring PTSS in pharmacists is still an understudied aspect of their pandemic experience.

Studies have shown reduced resilience levels among individuals who experience prolonged periods of work fatigue. Work fatigue is defined as a condition that has significant implications for employees' health, work performance, attitude, and safety [30,31]. It encompasses extreme exhaustion and tiredness experienced during and after workdays. Work fatigue can be categorized into three main types: physical, emotional, and mental. Physical work fatigue involves extreme physical tiredness and diminished physical capabilities. Emotional fatigue is characterized by emotional exhaustion and reduced capacity for emotional engagement during or after work. Mental work fatigue pertains to cognitive tiredness that hinders engagement in cognitive tasks during or after work [30,32]. Research conducted in various occupational settings, including pharmacists, has demonstrated that chronic workplace stress, excessive workload, and long working hours can reduce an individual's resilience [33–37]. This weakened resilience can manifest as increased vulnerability to stressors, impaired emotional regulation, and decreased problem-solving abilities [38,39]. On the other hand, there is evidence of a strong relationship between fatigue and the development of PTSS. Individuals who experience prolonged or severe fatigue, especially in the context of traumatic events such as the COVID-19 pandemic, are at an increased risk of developing PTSS [40]. This is evident in studies conducted on various populations, including military personnel, first responders, and healthcare workers, where those exposed to extreme fatigue due to long working hours, high stress, and overwhelming demands have shown a higher likelihood of developing PTSS [41–43].

It is therefore reasonable to hypothesize that work fatigue may interact with an individual's resilience, influencing its protective effect against PTSD. Specifically, It can be suggested that the presence of significant work fatigue may reduce the protective role of resilience, potentially making individuals more vulnerable to PTSD. In contrast, when work fatigue is minimal, resilience may have a more potent protective effect [44]. However, this complex interaction between resilience, work fatigue, and PTSD among pharmacists remains an area that needs plenty of research [45,46].

Resilience has been shown to be protective against adverse mental health outcomes when stress is present [47], unless their effects become weakened by moderating factors, such as work fatigue. Investigating possible moderators in the relationship between resilience and PTSD may pave the way for targeted interventions and developing support strategies that empower pharmacists to navigate the ongoing challenges of the pandemic while safeguarding their psychological well-being [48]. Therefore, the current study was conducted to investigate the role of work-related fatigue as a potential moderator in the relationship between pharmacists' resilience and their likelihood of experiencing PTSS during the COVID-19 pandemic. Specifically, the study aimed to explore whether the protective effect of resilience against PTSS is influenced or moderated by the levels of work-related fatigue experienced by pharmacists in multiple countries during this global health crisis.

**Methods**

Study design and sampling

A cross-sectional survey was conducted online in eight countries from January to December 2021, including Brazil, Lebanon, Nigeria, Pakistan, Poland, Serbia, and Tunisia. The choice of a multi-country design was made to provide a broader overview of the relation between study variables in various countries and regions of the world. The long period of data collection was due to the difficulty in availability of collaborators from the different countries involved, and in collecting data from pharmacists in the context of the COVID-19 situation, as this time of crisis substantially affected the daily practice of this population and required their priorities to be modified. This article represents an ongoing multinational collaboration on relevant public health topics between researchers from these countries.  The questionnaire was created using Google Forms, a cloud-based survey tool powered by Google™ and the questionnaire link was distributed online, using snowball sampling to enroll participants [49]. A convenient sample of at least 100 participants per country was set due to difficulties collecting data during the pandemic. Our samples consisted of pharmacists working in hospital or community pharmacy settings from eight countries. An introductory paragraph was included at the beginning of the link explaining the objectives of the study, while assuring participants about anonymity and confidentiality of their responses. After providing digital informed consent, participants were asked to complete a questionnaire. Participants completed the survey voluntarily and without remuneration.

Questionnaire

The online survey was formulated in English and translated to Arabic, Portuguese, Serbian and Polish according to the World Health Organization (WHO) translation guidelines. The research teams collaborated with a certified translator, consulted to detect discrepancies, solve them, validate and translate the questionnaire into the local language; this version was back-translated to English by a second certified translator in each country. A pilot test was done on 20 participants to make sure that the questions were well understood.

Sample size calculation

 To calculate the sample size, we used G*Power software. The minimum required sample size was 389 participants, considering an alpha error of 5%, a power of 80%, a minimal model r-square of 5%, and allowing 15 predictors for inclusion in the model.

Data collection

The first section of the questionnaire assessed the demographic characteristics, including age, gender, marital status, country of origin, primary work setting, the household crowding index, and financial distress or well-being scale.

The household crowding index is determined as follows: dividing the number of people by the number of rooms in the house except for the bathrooms and kitchen [50]. The InCharge Financial Distress/Financial Well-Being Scale is a set of 8 self-reported items representing the perceived financial well-being or distress status. Each response represents the participant financial state reflected on a continuum ranging from 1 (financial distress) to 10 (financial well-being) [51]. In this study, the Cronbach alpha value was 0.842.

The second section examined work exposure to COVID-19 and institutional support and demand during the pandemic. The third part intended to determine the sources of fear and factors contributing to resilience.

Outcomes measures

Four scales were used to assess the psychological impact of the COVID-19 pandemic on healthcare professionals.

1. Three-Dimensional Work Fatigue Inventory (3D-WFI)

The Three-Dimensional Work Fatigue Inventory (3D-WFI) is a validated measure for the evaluation of work fatigue [30,31]. According to Frone & Tidwel, work fatigue is a status of extreme tiredness and declined functional capacity to engage in physical, cognitive, and emotional activity experienced during and at the end of a work day [30]. The 3D-WFI consists of 18 items divided into three commensurate domains containing every six items, with three representing extreme tiredness and three others assessing reduced functional capacity. Respondents were provided a 5-point Likert scale to describe work fatigue during the past 12 months based on the three energetic resources (physical, mental and emotional). The scale ranged from never (score 0), less than once per month (score 1), at least once per month (score 2), at least once a week (score 3), and every day (score 4). Higher scores indicate higher work fatigue. In this study, Cronbach's alpha was 0.866, 0.887, and 0.905 for the physical, mental, and emotional subscales).

2. The Impact of Event scale (IES-6)

The Impact of Event scale (IES-6) [55] is an abbreviated version of the IES-R [56]. The IES-6 scales were widely used in the literature to assess the occurrence of PTSS due to COVID-19 [57–62]. The IES-6 scale consists of 6-items that represent three subscales describing the PTSS, such as avoidance (“I was aware that I still had a lot of feelings about it, but I didn't deal with them” and “I tried not to think about it”), intrusion (“Other things kept making me think about it” and “I thought about it when I did not mean to”), and hyperarousal (“I felt watchful or on guard” and “I had trouble concentrating”). Each subscale includes two items. The respondents were asked to indicate on a 5-point Likert scale their feelings during the COVID-19 pandemic before they got vaccinated. The Likert scale ranged from never (score 0), seldom (score 1), sometimes (score 2), often (score 3), and almost always (score 4). The summed score ranged from 0 to 24. Higher scores indicate higher PTSS symptoms. In this study, Cronbach's Alpha is 0.830.

3. Brief Resilience Scale (BRS)

The BRS is a tool for determining one ability to recover, cope, and bounce back after facing stressful events [63]. The BRS is a validated measure of resilience with a demonstrated Cronbach’s alpha ranging from 0.80 to 0.91 and a one-month test-retest reliability (ICC) of 0.69 [63]. It consists of six items, three positively and three negatively worded items. Respondents were provided a 5-point Likert scale to assess their resilience in facing the COVID-19 pandemic hurdles. The scale ranged from Strongly disagree (score 1) to strongly agree (score 5). The negative items (“I have a hard time making it through stressful events “, “It is hard for me to snap back when something bad happens” and “I tend to take a long time to get over set-backs in my life”) were scored by reverse coding. The total average score ranged from 1.00 to 5.00, where a value of 3.0–4.3 is considered a good level of resilience [64]. In this study, Cronbach's Alpha is 0.500).

**Statistical analysis**

The SPSS software v.26 was used for the statistical analysis. The IES score was considered normally distributed since the skewness (= .217) and kurtosis (= -.228) values varied between ±1. The Student *t* test was used to compare two means and the Pearson test to correlate two continuous variables. The moderation analysis was conducted using PROCESS MACRO (an SPSS add-on) v3.4 model 1, taking work fatigue as a moderator in the association between resilience and PTSD. Interaction terms were probed by examining the association of the predictor with PTSD at the mean, 1 SD below the mean and 1 SD above the mean of the moderators (physical/mental/emotional work fatigue). Results were adjusted over all variables that showed a *p* < .25 in the bivariate analysis. *P* < 0.05 was deemed statistically significant.

**Results**

A total of 442 pharmacists were enrolled in this study (mean age= 33.91 ± 10.36 years) with 59.5% of them being females. Other characteristics of the sample are shown in Table 1.

| **Table 1. Sociodemographic and other characteristics of the participants (n=442).** | |
| --- | --- |
| **Variable** | **n (%)** |
| **Country** |  |
| Developed | 46 (10.4%) |
| Developing | 396 (89.6%) |
| **Gender** |  |
| Male | 179 (40.5%) |
| Female | 263 (59.5%) |
| **Marital status** |  |
| Single / divorced | 285 (64.5%) |
| Married | 157 (35.5%) |
| **Specialty** |  |
| Hospital pharmacy | 206 (46.6%) |
| Community pharmacy | 236 (53.4%) |
| **Working in contact with COVID-19 patients** |  |
| No | 215 (48.6%) |
| Yes | 227 (51.4%) |
| **Working voluntarily hours** |  |
| No | 158 (35.7%) |
| Yes | 284 (64.3%) |
| **Working mandatory hours** |  |
| No | 301 (68.1%) |
| Yes | 141 (31.9%) |
|  | **Mean ± SD** |
| PTSD (IES scores) | 8.17 ± 4.82 |
| Resilience | 18.28 ± 3.78 |
| Physical work fatigue | 16.61 ± 4.99 |
| Mental work fatigue | 16.07 ± 5.12 |
| Emotional work fatigue | 15.32 ± 5.37 |
| Age (years) | 33.91 ± 10.36 |
| Household crowding index (person/room) | .85 ± .80 |
| Financial satisfaction | 37.10 ± 1257 |
| Number of months engaged in COVID | 7.55 ± 5.86 |
| Additional hours | 8.06 ± 13.95 |

**Bivariate analysis**

The bivariate analysis results are shown in Tables 2 and 3. A higher IES mean score was significantly found in females compared to males, and in participants who did not work mandatory hours. Furthermore, higher resilience was significantly associated with lower PTSD scores, whereas higher mental work fatigue was significantly associated with higher PTSD scores.

| **Table 2. Bivariate analysis of the categorical variables associated with PTSD.** | | | | |
| --- | --- | --- | --- | --- |
| **Variable** | **Mean ± SD** | ***t*** | ***df*** | ***p*** |
| **Country** |  | -1.199 | 440 | .231 |
| Developed | 8.08 ± 4.74 |  |  |  |
| Developing | 8.98 ± 5.45 |  |  |  |
| **Gender** |  | -2.564 | 440 | **.011** |
| Male | 7.46 ± 4.49 |  |  |  |
| Female | 8.65 ± 4.99 |  |  |  |
| **Marital status** |  | .001 | 440 | 1 |
| Single / divorced | 8.17 ± 4.96 |  |  |  |
| Married | 8.17 ± 4.58 |  |  |  |
| **Specialty** |  | .624 | 440 | .533 |
| Hospital pharmacy | 8.33 ± 4.67 |  |  |  |
| Community pharmacy | 8.04 ± 4.95 |  |  |  |
| **Working in contact with COVID-19 patients** |  | -1.357 | 440 | .176 |
| No | 7.85 ± 5.20 |  |  |  |
| Yes | 8.48 ± 4.42 |  |  |  |
| **Working voluntarily hours** |  | -1.326 | 440 | .186 |
| No | 7.73 ± 5.59 |  |  |  |
| Yes | 8.42 ± 4.33 |  |  |  |
| **Working mandatory hours** |  | 2.213 | 440 | **.028** |
| No | 8.54 ± 4.49 |  |  |  |
| Yes | 7.38 ± 5.40 |  |  |  |

Numbers in bold indicate significant *p* values.

| **Table 3. Correlation matrix of continuous variables.** | | | | | | | | | | |
| --- | --- | --- | --- | --- | --- | --- | --- | --- | --- | --- |
| **Variable** | **1** | **2** | **3** | **4** | **5** | **6** | **7** | **8** | **9** | **10** |
| 1. PTSD | 1 |  |  |  |  |  |  |  |  |  |
| 2. Resilience | -.19*** | 1 |  |  |  |  |  |  |  |  |
| 3. Physical work fatigue | -.04 | .02 | 1 |  |  |  |  |  |  |  |
| 4. Mental work fatigue | .12** | -.11* | .71*** | 1 |  |  |  |  |  |  |
| 5. Emotional work fatigue | .06 | -.16** | .63*** | .78*** | 1 |  |  |  |  |  |
| 6. Age | .09 | .001 | -.13** | -.13** | -.07 | 1 |  |  |  |  |
| 7. Household crowding index | -.09 | .09 | .12* | .12** | .08 | -.11* | 1 |  |  |  |
| 8. Financial satisfaction | .001 | .03 | -.31*** | -.26*** | -.33*** | .18*** | -.06 | 1 |  |  |
| 9. Number of months engaged in COVID | .09 | .15** | .10* | .08 | .02 | .17*** | -.05 | .01 | 1 |  |
| 10. Additional hours | .06 | -.03 | .12* | .13** | .06 | .06 | -.04 | -.12* | .14** | 1 |

Numbers in the table reflect Pearson correlation coefficients; ***p* <.01; ****p* <.001

**Moderation analysis with psychological distress taken as the dependent variable**

The details of the moderation analysis of work fatigue taken as a moderator in the association between resilience and PTSD, are summarized in Table 4. The results were adjusted over country, gender, working in contact with COVID-19, working patients, working mandatory hours, working voluntary hours, age, household crowding index and number of months engaged in COVID-19. The interactions resilience by physical (Beta = .02; *p* = .029), mental (Beta = .02; *p* = .040) and emotional (Beta = .03; *p* = .008) work fatigue were significantly associated with PTSD (Table 4); for pharmacists with low to moderate levels of physical (Beta = -.33; *p* < .001 and Beta = -.21; p = .001), mental (Beta = -.29; *p* < .001 and Beta = -.18; p = .006) and emotional (Beta = -.31; *p* < .001 and Beta = -.17; p = .008) work fatigue, higher resilience was significantly related to lower PTSD levels. For pharmacists with high levels of physical/mental/emotional work fatigue, the association between resilience and PTSD became non-significant (Table 5).

| **Table 4. Moderation analysis taking resilience as the independent variable, work fatigue subscales as moderators and PTSD scores as the dependent variable.** | | | | |
| --- | --- | --- | --- | --- |
| **Model 1: Physical work fatigue as the moderator.** | | | | |
|  | **Beta** | ***t*** | ***P*** | **95% CI** |
| Resilience | -.61 | -3.263 | **.001** | -.98; -.24 |
| Physical work fatigue | -.49 | -2.254 | **.025** | -.92; -.06 |
| Interaction resilience by physical work fatigue | .02 | 2.196 | **.029** | .003; .046* |
| **Model 2: Mental work fatigue as the moderator.** | | | | |
| Resilience | -.52 | -3.125 | **.002** | -.84; -.19 |
| Mental work fatigue | -.30 | -1.434 | .152 | -.70; .11 |
| Interaction resilience by mental work fatigue | .02 | 2.058 | **.040** | .001; .041* |
| **Model 3: Emotional work fatigue as the moderator.** | | | | |
| Resilience | -.57 | -3.836 | **<.001** | -.86; -.28 |
| Emotional work fatigue | -.46 | -2.377 | **.018** | -.84; -.08 |
| Interaction resilience by emotional work fatigue | .03 | 2.649 | **.008** | .01; .05* |

*indicates significant moderation; numbers in bold indicate significant *p* values.

| **Table 5. Conditional effects of the focal predictor (resilience) at values of the moderator (work fatigue).** | | | | |
| --- | --- | --- | --- | --- |
|  | **Beta** | ***t*** | ***p*** | **95% CI** |
| **Model 1: Physical work fatigue as the moderator.** | | | | |
| Low (= 11.61) | -.33 | -4.17 | **<.001** | -.48; -.17 |
| Moderate (= 16.59) | -.21 | -3.24 | **.001** | -.33; -.08 |
| High (= 21.58) | -.08 | -.93 | .354 | -.26; .09 |
| **Model 2: Mental work fatigue as the moderator.** | | | | |
| Low (= 10.95) | -.29 | -3.81 | **<.001** | -.43; -.14 |
| Moderate (= 16.08) | -.18 | -2.79 | **.006** | -.30; -.05 |
| High (= 21.20) | -.07 | -.79 | .432 | -.25; .11 |
| **Model 3: Emotional work fatigue as the moderator.** | | | | |
| Low (= 9.93) | -.31 | -4.24 | **<.001** | -.46; -.17 |
| Moderate (= 15.31) | -.17 | -2.65 | **.008** | -.30; -.04 |
| High (= 20.69) | -.03 | -.36 | .716 | -.22; .15 |

Numbers in bold indicate significant *p* values.

**Discussion**

The COVID-19 pandemic has posed unprecedented challenges to healthcare professionals across the globe, and pharmacists, as essential frontline workers, have been no exception. The multifaceted demands placed on pharmacists during this crisis have been accompanied by high prevalence rates of PTSS [65,66] and low levels of resilience [27,67]. Against this backdrop, this cross-sectional multi-country study was conducted to delve into the intricate interplay between resilience and PTSS among pharmacists during the COVID-19 pandemic. Specifically, it was investigated how varying levels of work fatigue, stemming from the physical, mental, and emotional toll of their profession, may moderate the relationship between resilience and PTSS. Our results showed that in pharmacists experiencing mild to moderate physical, mental, and emotional work fatigue, greater resilience was strongly linked to reduced levels of PTSS. However, for pharmacists facing high levels of physical, mental, or emotional work fatigue, the connection between resilience and PTSS did not show statistical significance. These findings not only address a critical gap in our understanding of pharmacist well-being during a global health crisis, but also offer insights that can inform targeted interventions aimed at bolstering the mental health and resilience of these vital healthcare professionals.

One of the findings of this study pertain to pharmacists’ PTSS levels in relation to resilience. A robust and significant association was found between higher levels of resilience and lower PTSS scores, suggesting that pharmacists exhibiting higher levels of resilience were less likely to report PTSS. This finding is expected, on the basis of the well-established protective role of resilience in buffering against the psychological toll of high-stress situations [68–71]. Pharmacists who displayed greater resilience exhibited a remarkable ability to cope with the demands and uncertainties of their profession during this crisis, resulting in reduced PTSS. In a prior investigation carried out in Lebanon, it was found that community pharmacists exhibited a relatively low level of resilience during the COVID-19 pandemic, and this was associated with elevated rates of burnout [27]. Another study conducted also on Qatari pharmacists during the pandemic revealed that they encountered a moderate level of burnout, yet they exhibited a moderate level of resilience, suggesting their capacity to effectively cope with challenges [67].

Additionally, our findings also unveiled a compelling relationship between higher mental work fatigue and elevated PTSS scores among pharmacists. This result implies that pharmacists who experienced heightened mental fatigue, possibly stemming from the complexity and volume of their tasks, such as navigating complex medication interactions or managing a high number of prescriptions, took on the mental well-being of these healthcare professionals. The mental fatigue associated with their professional responsibilities during the pandemic seemed to amplify the impact of stressors on their psychological well-being. Interviews with pharmacists in Wisconsin showed that they grappled with mental exhaustion, particularly on days marked by heavier workloads. This mental fatigue had an impact on their interactions with both fellow staff members, patients, and family members [72]. Additionally, another study showed that most pharmacists were identified with moderate-high likelihood of burnout and moderate-high probability of secondary traumatic stress at rates which are significantly higher compared with rates early in the pandemic [73].

Moreover, a moderation analysis was conducted in this study to examine the moderating role of work-related fatigue on the relationship between resilience and PTSS. This approach provides valuable insights into the complex interplay between personal resilience, work-related fatigue, and post-traumatic stress outcomes, shedding light on potential factors that could mitigate or exacerbate the effects of resilience on PTSS in occupational settings.

Our analysis demonstrated a significant correlation between increased resilience and reduced PTSS levels among pharmacists who experienced low to moderate levels of physical, mental, and emotional fatigue in their work. However, the association between resilience and PTSD became non-significant for pharmacists with high levels of the three work fatigue dimensions. In essence, when pharmacists exhibit greater resilience and operate within the bounds of manageable work-related fatigue, they tend to report lower levels of PTSS. This suggests that resilience may function as a protective mechanism, mitigating the impact of work-related challenges on their mental health, especially when the fatigue associated with their professional duties remains within reasonable limits. On the other hand, when pharmacists experienced high levels of physical and mental fatigue due to their job demands, the buffering effect of resilience on PTSS appears to diminish or weaken. In such cases, the impact of work fatigue may overwhelm the potential protective influence of resilience. An abundance of research has focused on identifying factors affecting mental health in pharmacists [8,17,66], however there is a paucity of research examining the moderating effect of work fatigue on the association between resilience and PTSS among them. Therefore, it was not feasible to compare our study results with similar research because there was a scarcity of existing studies on the same topic. Nevertheless, numerous studies link resilience with burnout, fatigue and stress in HCWs [74–77]. In a prior longitudinal study, it was determined that resilience had a mitigating effect on nurse fatigue [78]. Another study involving healthcare professionals found that individuals with higher levels of resilience reported improved perceived immune function and a reduction in both physical and psychological symptom [79]. Also, multiple studies conducted to explore the relationship between exhaustion and PTSS resulting from excessive workplace stress have consistently demonstrated a robust and positive correlation between burnout and PTSS [80–82].

***Clinical Implications***

The current study carries significant implications for both the pharmacy profession and the broader healthcare sector. The findings shed light on the crucial role of resilience in mitigating the psychological impact of a global health crisis on healthcare workers. Recognizing the role of resilience in buffering the impact of work-related stressors on mental health and reducing the development of PTSS highlights the need for resilience-building interventions and support systems within the pharmaceutical field to enhance pharmacists' mental well-being during such crises. Furthermore, the identification of work fatigue as a moderator underscores the importance of managing fatigue and workload through implementing fatigue management strategies, optimizing work hours, and prioritizing the well-being of these professionals to help safeguard their resilience and reduce the risk of developing PTSS. These insights can inform policy decisions and workplace strategies to better protect the mental health of pharmacists and other frontline HCWs during and beyond pandemics, ultimately ensuring their ability to provide quality care to patients.

***Strengths and Limitations***

The present study possesses several notable strengths and limitations. A key strength lies in its multi-country approach, which enhances the generalizability of findings offering a broad perspective on the relationship between resilience, work fatigue, and PTSS among pharmacists during the COVID-19 pandemic. Furthermore, its timeliness and relevance to current events provide valuable insights into the unique challenges faced by pharmacists and healthcare professionals during a critical period. The utilization of resilience as a variable and the study's quantitative approach strengthens the empirical foundation of the study. However, several limitations should be acknowledged. The cross-sectional design of the study restricts the ability to determine causality, and reliance on self-reported data introduces potential bias. Subjects were recruited from January to December 2021; therefore, participants' conditions might have varied depending on the time of the survey. The quality of the survey may not be high in online surveys; in addition, we could not know which participants answered the survey in an inappropriate way to exclude them. Additionally, the generalizability of findings to other professions may be limited, and the study's focus on a specific set of variables leaves other potential contributors to pharmacists' mental well-being unexplored. Despite these limitations, the study significantly contributes to our understanding of the mental health dynamics faced by pharmacists in high-stress contexts like the COVID-19 pandemic and underscores the need for future research to delve further into the complexities of this issue, especially during times of heightened stress and crisis.

**Conclusion**

In summary, this study highlights the intricate interplay between resilience, work-related fatigue, and PTSS levels among pharmacists. It highlights the role of resilience in reducing PTSS and underscore the imperative of addressing mental work fatigue to safeguard the psychological health of pharmacists during challenging times. It offers a nuanced perspective on the factors influencing mental well-being among pharmacists during times of crisis, providing valuable insights for tailored support and interventions that not only foster resilience-building strategies but also address the unique challenges of work fatigue within the pharmacy profession.

**DECLARATIONS**

**Ethics Approval and Consent to Participate:**

The study protocol was approved by the ethics committee of the School of Pharmacy at the Lebanese International University (Reference # 2020RC-058-LIUSOP). All collaborators who collected data were asked to follow the ethical guidelines of their Institutional Review Board (IRB), acting either on the ethical approval of the Principal Investigator (KI) or that received from their local IRBs. Written informed consent was obtained from all subjects for study participation; the online submission of the soft copy was considered equivalent to receiving a written informed consent. All methods were carried out in accordance with relevant guidelines and regulations.

**Consent for publication:**

Not applicable.

**Availability of data and materials:**

All data generated or analyzed during this study are not publicly available due the restrictions from the ethics committee. Reasonable requests can be addressed to the corresponding author (SH).

**Competing interests:**

The authors have nothing to disclose.

**Funding:**

None.

**Author contributions:**

FFR, SH and KI designed the study; SY drafted the manuscript; KI, SH, IM, SK, ABM, SCE, KE, RJ, NP, PS, MC, SY, MA, RC collected the data; SH carried out the analysis and interpreted the results; KI, SH, FFR, IM, SK, ABM, SCE, KE, RJ, NP, PS, MC, SY, MA, RC reviewed the paper for intellectual content; all authors reviewed the final manuscript and gave their consent.

**Acknowledgements:** The authors would like to thank all participants.

**References**

1. Cadogan, C.A.; Hughes, C.M. On the Frontline against COVID-19: Community Pharmacists’ Contribution during a Public Health Crisis. *Res Social Adm Pharm* **2021**, *17*, 2032–2035, doi:10.1016/j.sapharm.2020.03.015.

2. Bukhari, N.; Rasheed, H.; Nayyer, B.; Babar, Z.-U.-D. Pharmacists at the Frontline Beating the COVID-19 Pandemic. *Journal of Pharmaceutical Policy and Practice* **2020**, doi:10.1186/s40545-020-00210-w.

3. Alves da Costa, F.; Lee, V.; Leite, S.N.; Murillo, M.D.; Menge, T.; Antoniou, S. Pharmacists Reinventing Their Roles to Effectively Respond to COVID-19: A Global Report from the International Pharmacists for Anticoagulation Care Taskforce (iPACT). *Journal of Pharmaceutical Policy and Practice* **2020**, *13*, 12, doi:10.1186/s40545-020-00216-4.

4. Visacri, M.B.; Figueiredo, I.V.; Lima, T. de M. Role of Pharmacist during the COVID-19 Pandemic: A Scoping Review. *Res Social Adm Pharm* **2021**, *17*, 1799–1806, doi:10.1016/j.sapharm.2020.07.003.

5. Dos Santos, P.M.; da Silva, C.R.; Costa, D.; Torre, C. Burnout in the Pharmaceutical Activity: The Impact of COVID-19. *Front Psychiatry* **2021**, *12*, 771462, doi:10.3389/fpsyt.2021.771462.

6. Elbeddini, A.; Prabaharan, T.; Almasalkhi, S.; Tran, C. Pharmacists and COVID-19. *Journal of Pharmaceutical Policy and Practice* **2020**, *13*, doi:10.1186/s40545-020-00241-3.

7. Visacri, M.B.; Figueiredo, I.V.; Lima, T. de M. Role of Pharmacist during the COVID-19 Pandemic: A Scoping Review. *Res Social Adm Pharm* **2021**, *17*, 1799–1806, doi:10.1016/j.sapharm.2020.07.003.

8. Elbeddini, A.; Wen, C.X.; Tayefehchamani, Y.; To, A. Mental Health Issues Impacting Pharmacists during COVID-19. *Journal of Pharmaceutical Policy and Practice* **2020**, *13*, 46, doi:10.1186/s40545-020-00252-0.

9. Carmassi, C.; Foghi, C.; Dell’Oste, V.; Cordone, A.; Bertelloni, C.A.; Bui, E.; Dell’Osso, L. PTSD Symptoms in Healthcare Workers Facing the Three Coronavirus Outbreaks: What Can We Expect after the COVID-19 Pandemic. *Psychiatry Res* **2020**, *292*, 113312, doi:10.1016/j.psychres.2020.113312.

10. Horesh, D.; Brown, A.D. Traumatic Stress in the Age of COVID-19: A Call to Close Critical Gaps and Adapt to New Realities. *Psychol Trauma* **2020**, *12*, 331–335, doi:10.1037/tra0000592.

11. *Diagnostic and Statistical Manual of Mental Disorders: DSM-5-TR*; American Psychiatric Association Publishing, 2022; ISBN 978-0-89042-578-7.

12. Preti, E.; Di Mattei, V.; Perego, G.; Ferrari, F.; Mazzetti, M.; Taranto, P.; Di Pierro, R.; Madeddu, F.; Calati, R. The Psychological Impact of Epidemic and Pandemic Outbreaks on Healthcare Workers: Rapid Review of the Evidence. *Curr Psychiatry Rep* **2020**, *22*, 43, doi:10.1007/s11920-020-01166-z.

13. Andhavarapu, S.; Yardi, I.; Bzhilyanskaya, V.; Lurie, T.; Bhinder, M.; Patel, P.; Pourmand, A.; Tran, Q.K. Post-Traumatic Stress in Healthcare Workers during the COVID-19 Pandemic: A Systematic Review and Meta-Analysis. *Psychiatry Res* **2022**, *317*, 114890, doi:10.1016/j.psychres.2022.114890.

14. Benfante, A.; Di Tella, M.; Romeo, A.; Castelli, L. Traumatic Stress in Healthcare Workers During COVID-19 Pandemic: A Review of the Immediate Impact. *Front Psychol* **2020**, *11*, 569935, doi:10.3389/fpsyg.2020.569935.

15. d’Ettorre, G.; Ceccarelli, G.; Santinelli, L.; Vassalini, P.; Innocenti, G.P.; Alessandri, F.; Koukopoulos, A.E.; Russo, A.; d’Ettorre, G.; Tarsitani, L. Post-Traumatic Stress Symptoms in Healthcare Workers Dealing with the COVID-19 Pandemic: A Systematic Review. *Int J Environ Res Public Health* **2021**, *18*, 601, doi:10.3390/ijerph18020601.

16. Marvaldi, M.; Mallet, J.; Dubertret, C.; Moro, M.R.; Guessoum, S.B. Anxiety, Depression, Trauma-Related, and Sleep Disorders among Healthcare Workers during the COVID-19 Pandemic: A Systematic Review and Meta-Analysis. *Neurosci Biobehav Rev* **2021**, *126*, 252–264, doi:10.1016/j.neubiorev.2021.03.024.

17. Ishaky, L.; Sivanthan, M.; Tadrous, M.; Nowrouzi-Kia, B.; McCarthy, L.; Papadopoulos, A.; Gohar, B. Pharmacists’ Mental Health during the First Two Years of the Pandemic: A Socio-Ecological Scoping Review. *Pharmacy (Basel)* **2023**, *11*, 64, doi:10.3390/pharmacy11020064.

18. Jones, A.M.; Clark, J.S.; Mohammad, R.A. Burnout and Secondary Traumatic Stress in Health-System Pharmacists during the COVID-19 Pandemic. *American Journal of Health-System Pharmacy* **2021**, *78*, 818–824, doi:10.1093/ajhp/zxab051.

19. Alshehri, A.S.; Alghamdi, A.H. Post-Traumatic Stress Disorder Among Healthcare Workers Diagnosed With COVID-19 in Jeddah, Kingdom of Saudi Arabia, 2020 to 2021. *Cureus* **2021**, *13*, e17371, doi:10.7759/cureus.17371.

20. Colón-López, A.; Meese, K.A.; Montgomery, A.P.; Patrician, P.A.; Rogers, D.A.; Burkholder, G.A. Unique Stressors in a Global Pandemic: A Mixed Methods Study about Unique Causes of Distress among Healthcare Team Members during COVID-19. *Journal of Hospital Management and Health Policy* **2022**, *6*, doi:10.21037/jhmhp-21-69.

21. He, Q.; Xu, P.; Wang, H.; Wang, S.; Yang, L.; Ba, Z.; Huang, H. The Mediating Role of Resilience between Perceived Social Support and Sense of Security in Medical Staff Following the COVID-19 Pandemic: A Cross-Sectional Study. *Frontiers in Psychiatry* **2023**, *14*.

22. Sisto, A.; Vicinanza, F.; Campanozzi, L.L.; Ricci, G.; Tartaglini, D.; Tambone, V. Towards a Transversal Definition of Psychological Resilience: A Literature Review. *Medicina (Kaunas)* **2019**, *55*, 745, doi:10.3390/medicina55110745.

23. Bajis, D.; Bajis, S.; Akel, M.; Bizama, A.P.; Chaar, B. A Grounded Theory Approach to Exploring the Experiences of Community Pharmacists in Lebanon to a Triple Whammy of Crises: The Lebanese Financial Crisis, COVID-19 Pandemic, and the Beirut Port Explosion. *Explor Res Clin Soc Pharm* **2023**, *9*, 100217, doi:10.1016/j.rcsop.2022.100217.

24. Wrenn, D.G.L.; Wingo, D.A.P.; Moore, D.R.; Pelletier, M.T.; Gutman, D.A.R.; Bradley, D.B.; Ressler, D.K.J. The Effect of Resilience on Posttraumatic Stress Disorder in Trauma-Exposed Inner-City Primary Care Patients. *Journal of the National Medical Association* **2011**, *103*, 560, doi:10.1016/s0027-9684(15)30381-3.

25. Vg, S.; Sm, A.; M, D. Associations between Changes in Resilient Coping and Posttraumatic Stress Disorder Symptoms. *Research in nursing & health* **2020**, *43*, doi:10.1002/nur.22014.

26. Wu, K.; Zhang, Y.; Liu, Z.; Zhou, P.; Wei, C. Coexistence and Different Determinants of Posttraumatic Stress Disorder and Posttraumatic Growth among Chinese Survivors after Earthquake: Role of Resilience and Rumination. *Frontiers in Psychology* **2015**, *6*.

27. Alameddine, M.; Bou-Karroum, K.; Hijazi, M.A. A National Study on the Resilience of Community Pharmacists in Lebanon: A Cross-Sectional Survey. *Journal of Pharmaceutical Policy and Practice* **2022**, *15*, 8, doi:10.1186/s40545-022-00406-2.

28. Epp, D.A.; Fujii, Y.; Shiratani, T. A Study of Pharmacists’ Resilience-Enhancing Behaviours to Improve Pharmacy Student Resiliency in Japan. *Pharmacy Education* **2022**, *22*, 715–726, doi:10.46542/pe.2022.221.715726.

29. Ss, W.; L, W.; R, C.; Mj, R.; Jd, C. The Relationship Between Pharmacist Resilience, Burnout, and Job Performance. *Journal of pharmacy practice* **2023**, doi:10.1177/08971900231164886.

30. Frone, M.R.; Tidwell, M.-C.O. The Meaning and Measurement of Work Fatigue: Development and Evaluation of the Three-Dimensional Work Fatigue Inventory (3D-WFI). *J Occup Health Psychol* **2015**, *20*, 273–288, doi:10.1037/a0038700.

31. Sfeir, E.; Rabil, J.-M.; Obeid, S.; Hallit, S.; Khalife, M.-C.F. Work Fatigue among Lebanese Physicians and Students during the COVID-19 Pandemic: Validation of the 3D-Work Fatigue Inventory (3D-WFI) and Correlates. *BMC Public Health* **2022**, *22*, 292, doi:10.1186/s12889-022-12733-9.

32. Blais, A.-R.; Gillet, N.; Houle, S.A.; Comeau, C.A.; Morin, A.J.S. Work Fatigue Profiles: Nature, Implications, and Associations With Psychological Empowerment. *Front Psychol* **2020**, *11*, 596206, doi:10.3389/fpsyg.2020.596206.

33. Bhui, K.; Dinos, S.; Galant-Miecznikowska, M.; de Jongh, B.; Stansfeld, S. Perceptions of Work Stress Causes and Effective Interventions in Employees Working in Public, Private and Non-Governmental Organisations: A Qualitative Study. *BJPsych Bull* **2016**, *40*, 318–325, doi:10.1192/pb.bp.115.050823.

34. Birhanu, M.; Gebrekidan, B.; Tesefa, G.; Tareke, M. Workload Determines Workplace Stress among Health Professionals Working in Felege-Hiwot Referral Hospital, Bahir Dar, Northwest Ethiopia. *J Environ Public Health* **2018**, *2018*, 6286010, doi:10.1155/2018/6286010.

35. Langran, C.; Mantzourani, E.; Hughes, L.; Hall, K.; Willis, S. “I’m at Breaking Point”; Exploring Pharmacists’ Resilience, Coping and Burnout during the COVID-19 Pandemic. *Explor Res Clin Soc Pharm* **2022**, *5*, 100104, doi:10.1016/j.rcsop.2022.100104.

36. Piotrowski, A.; Sygit-Kowalkowska, E.; Boe, O.; Rawat, S. Resilience, Occupational Stress, Job Satisfaction, and Intention to Leave the Organization among Nurses and Midwives during the COVID-19 Pandemic. *Int J Environ Res Public Health* **2022**, *19*, 6826, doi:10.3390/ijerph19116826.

37. Schommer, J.C.; Gaither, C.A.; Alvarez, N.A.; Lee, S.; Shaughnessy, A.M.; Arya, V.; Planas, L.G.; Fadare, O.; Witry, M.J. Pharmacy Workplace Wellbeing and Resilience: Themes Identified from a Hermeneutic Phenomenological Analysis with Future Recommendations. *Pharmacy (Basel)* **2022**, *10*, 158, doi:10.3390/pharmacy10060158.

38. Dai, Q.; Smith, G.D. Resilience to Depression: Implication for Psychological Vaccination. *Front Psychiatry* **2023**, *14*, 1071859, doi:10.3389/fpsyt.2023.1071859.

39. Faye, C.; McGowan, J.C.; Denny, C.A.; David, D.J. Neurobiological Mechanisms of Stress Resilience and Implications for the Aged Population. *Current Neuropharmacology* **2018**, *16*, 234, doi:10.2174/1570159X15666170818095105.

40. Zeng, F.; John, W.C.M.; Sun, X.; Wang, Y. COVID-19-Associated Impact and Post-Traumatic Stress Symptoms 39 Days after Pandemic in a Sample of Home-Quarantined Chinese College Students: The Mediating Effecting of Past Stressful Events, Psychological Resilience, and Social Support. *BMC Psychiatry* **2023**, *23*, 379, doi:10.1186/s12888-023-04906-6.

41. Cocker, F.; Joss, N. Compassion Fatigue among Healthcare, Emergency and Community Service Workers: A Systematic Review. *Int J Environ Res Public Health* **2016**, *13*, 618, doi:10.3390/ijerph13060618.

42. Herraiz-Recuenco, L.; Alonso-Martínez, L.; Hannich-Schneider, S.; Puente-Alcaraz, J. Causes of Stress among Healthcare Professionals and Successful Hospital Management Approaches to Mitigate It during the COVID-19 Pandemic: A Cross-Sectional Study. *Int J Environ Res Public Health* **2022**, *19*, 12963, doi:10.3390/ijerph191912963.

43. Lawn, S.; Roberts, L.; Willis, E.; Couzner, L.; Mohammadi, L.; Goble, E. The Effects of Emergency Medical Service Work on the Psychological, Physical, and Social Well-Being of Ambulance Personnel: A Systematic Review of Qualitative Research. *BMC Psychiatry* **2020**, *20*, 348, doi:10.1186/s12888-020-02752-4.

44. Labrague, L.J.; de los Santos, J.A.A. Resilience as a Mediator between Compassion Fatigue, Nurses’ Work Outcomes, and Quality of Care during the COVID-19 Pandemic. *Appl Nurs Res* **2021**, *61*, 151476, doi:10.1016/j.apnr.2021.151476.

45. Stoewen, D.L. Moving from Compassion Fatigue to Compassion Resilience Part 6: Building Organizational Resilience. *Can Vet J* **2022**, *63*, 203–205.

46. Zarowsky, Z.; Rashid, T. Resilience and Wellbeing Strategies for Pandemic Fatigue in Times of Covid-19. *Int J Appl Posit Psychol* **2023**, *8*, 1–36, doi:10.1007/s41042-022-00078-y.

47. Ozbay, F.; Johnson, D.C.; Dimoulas, E.; Morgan, C.A.; Charney, D.; Southwick, S. Social Support and Resilience to Stress. *Psychiatry (Edgmont)* **2007**, *4*, 35–40.

48. Schwartz, R.M.; McCann-Pineo, M.; Bellehsen, M.; Singh, V.; Malhotra, P.; Rasul, R.; Corley, S.S.; Jan, S.; Parashar, N.; George, S.; et al. The Impact of Physicians’ COVID-19 Pandemic Occupational Experiences on Mental Health. *J Occup Environ Med* **2022**, *64*, 151–157, doi:10.1097/JOM.0000000000002380.

49. Parker, C.; Scott, S.; Geddes, A. *Snowball Sampling*; SAGE Publications Ltd, 2019; ISBN 978-1-5297-4761-4.

50. Melki, I.; Beydoun, H.; Khogali, M.; Tamim, H.; Yunis, K. Household Crowding Index: A Correlate of Socioeconomic Status and Inter-Pregnancy Spacing in an Urban Setting. *J Epidemiol Community Health* **2004**, *58*, 476–480, doi:10.1136/jech.2003.012690.

51. Prawitz, A.D.; Garman, E.T.; Sorhaindo, B.; O’Neill, B.; Kim, J.; Drentea, P. Incharge Financial Distress/Financial Well-Being Scale: Development, Administration, and Score Interpretation. *Financial Counseling and Planning* **2006**, *17*, 34–50.

52. Lovibond, P.F.; Lovibond, S.H. The Structure of Negative Emotional States: Comparison of the Depression Anxiety Stress Scales (DASS) with the Beck Depression and Anxiety Inventories. *Behaviour Research and Therapy* **1995**, *33*, 335–343, doi:10.1016/0005-7967(94)00075-U.

53. Henry, J.D.; Crawford, J.R. The Short-Form Version of the Depression Anxiety Stress Scales (DASS-21): Construct Validity and Normative Data in a Large Non-Clinical Sample. *British Journal of Clinical Psychology* **2005**, *44*, 227–239, doi:10.1348/014466505X29657.

54. Shekhar, S.; Ahmad, S.; Ranjan, A.; Pandey, S.; Ayub, A.; Kumar, P. Assessment of Depression, Anxiety and Stress Experienced by Health Care and Allied Workers Involved in SARS-CoV2 Pandemic. *J Family Med Prim Care* **2022**, *11*, 466–471, doi:10.4103/jfmpc.jfmpc_2518_20.

55. Thoresen, S.; Tambs, K.; Hussain, A.; Heir, T.; Johansen, V.A.; Bisson, J.I. Brief Measure of Posttraumatic Stress Reactions: Impact of Event Scale-6. *Soc Psychiatry Psychiatr Epidemiol* **2010**, *45*, 405–412, doi:10.1007/s00127-009-0073-x.

56. Weiss, D.S. The Impact of Event Scale: Revised. In *Cross-cultural assessment of psychological trauma and PTSD*; International and cultural psychology; Springer Science + Business Media: New York, NY, US, 2007; pp. 219–238 ISBN 978-0-387-70989-5.

57. Horn, M.; Wathelet, M.; Fovet, T.; Amad, A.; Vuotto, F.; Faure, K.; Astier, T.; Noël, H.; Duhem, S.; Vaiva, G.; et al. Is COVID-19 Associated With Posttraumatic Stress Disorder? *J Clin Psychiatry* **2020**, *82*, 20m13641, doi:10.4088/JCP.20m13641.

58. Dutta, P.; Anand, S.; Gupta, S.; Kanchan, R.K.; Parhi, K.K. Assessment of Post-Traumatic Stress Disorder Symptoms during COVID-19 Pandemic among Medical Students. *Biomedicine (India)* **2022**, 117–121.

59. Jeong, J.; Kim, A.-R.; Hilton, C.; Hong, I. Impact of Event Scale-6 (IES-6) for U.S. Adults Who Experienced the COVID-19 Pandemic. *BMC Psychiatry* **2022**, *22*, 490, doi:10.1186/s12888-022-04136-2.

60. Vanaken, L.; Scheveneels, S.; Belmans, E.; Hermans, D. Validation of the Impact of Event Scale With Modifications for COVID-19 (IES-COVID19). *Front Psychiatry* **2020**, *11*, 738, doi:10.3389/fpsyt.2020.00738.

61. Szepietowska, E.M.; Zawadzka, E.; Filipiak, S. Symptoms of Post-Traumatic Stress Disorder and the Sense of Gains and Losses during the COVID-19 Pandemic: An International Study. *International Journal of Environmental Research and Public Health* **2022**, *19*, 3504, doi:10.3390/ijerph19063504.

62. Si, M.-Y.; Su, X.-Y.; Jiang, Y.; Wang, W.-J.; Gu, X.-F.; Ma, L.; Li, J.; Zhang, S.-K.; Ren, Z.-F.; Ren, R.; et al. Psychological Impact of COVID-19 on Medical Care Workers in China. *Infect Dis Poverty* **2020**, *9*, 113, doi:10.1186/s40249-020-00724-0.

63. Smith, B.W.; Dalen, J.; Wiggins, K.; Tooley, E.; Christopher, P.; Bernard, J. The Brief Resilience Scale: Assessing the Ability to Bounce Back. *Int J Behav Med* **2008**, *15*, 194–200, doi:10.1080/10705500802222972.

64. Smith, B.; Epstein, E.; Ortiz, J.; Christopher, P.; Tooley, E. The Foundation of Resilience: What Are the Critical Resources For Bouncing Back From Stress? In; 2013; pp. 167–187 ISBN 978-1-4614-4938-6.

65. Lange, M.; Joo, S.; Couette, P.-A.; de Jaegher, S.; Joly, F.; Humbert, X. Impact on Mental Health of the COVID-19 Outbreak among Community Pharmacists during the Sanitary Lockdown Period. *Ann Pharm Fr* **2020**, *78*, 459–463, doi:10.1016/j.pharma.2020.09.002.

66. Safwan, J.; Halat, D.H.; Akel, M.; Younes, S.; Rahal, M.; Mourad, N.; Akiki, Z.; Cherfane, M.; Saade, F.; Bouraad, E.; et al. The Impact of COVID-19 on the Mental Health of Lebanese Pharmacists: A National Cross-Sectional Study. *Front Public Health* **2023**, *11*, 1156840, doi:10.3389/fpubh.2023.1156840.

67. Samir AlKudsi, Z.; Hany Kamel, N.; El-Awaisi, A.; Shraim, M.; Saffouh El Hajj, M. Mental Health, Burnout and Resilience in Community Pharmacists during the COVID-19 Pandemic: A Cross-Sectional Study. *Saudi Pharm J* **2022**, *30*, 1009–1017, doi:10.1016/j.jsps.2022.04.015.

68. Gilbar, O.; Gelkopf, M.; Greene, T. Perceived Stress during COVID-19: Community Resilience Three Years before the Pandemic as a Protective Factor. *International Journal of Disaster Risk Reduction* **2022**, *82*, 103337, doi:10.1016/j.ijdrr.2022.103337.

69. Guillasper, J.N.; Oducado, R.M.F.; Soriano, G.P. Protective Role of Resilience on COVID-19 Impact on the Quality of Life of Nursing Students in the Philippines. *Belitung Nurs J* **2021**, *7*, 43–49, doi:10.33546/bnj.1297.

70. Havnen, A.; Anyan, F.; Hjemdal, O.; Solem, S.; Gurigard Riksfjord, M.; Hagen, K. Resilience Moderates Negative Outcome from Stress during the COVID-19 Pandemic: A Moderated-Mediation Approach. *Int J Environ Res Public Health* **2020**, *17*, 6461, doi:10.3390/ijerph17186461.

71. Padmanabhanunni, A.; Pretorius, T.B.; Khamisa, N. The Role of Resilience in the Relationship between Role Stress and Psychological Well-Being during the COVID-19 Pandemic: A Cross-Sectional Study. *BMC Psychology* **2023**, *11*, 45, doi:10.1186/s40359-023-01082-w.

72. Watterson, T.L.; Chui, M.A. Subjective Perceptions of Occupational Fatigue in Community Pharmacists. *Pharmacy* **2023**, *11*, 84, doi:10.3390/pharmacy11030084.

73. Mohammad, R.A.; Jones, A.M.; Clark, J.S. Changing Patterns of the Prevalence of Burnout and Secondary Traumatic Stress in Health‐system Pharmacists throughout the COVID ‐19 Pandemic. *J Am Coll Clin Pharm* **2022**, *5*, 674–681, doi:10.1002/jac5.1632.

74. Gonçalves, L.; Sala, R.; Navarro, J.-B. Resilience and Occupational Health of Health Care Workers: A Moderator Analysis of Organizational Resilience and Sociodemographic Attributes. *Int Arch Occup Environ Health* **2022**, *95*, 223–232, doi:10.1007/s00420-021-01725-8.

75. Lebares, C.C.; Guvva, E.V.; Ascher, N.L.; O’Sullivan, P.S.; Harris, H.W.; Epel, E.S. Burnout and Stress Among US Surgery Residents: Psychological Distress and Resilience. *J Am Coll Surg* **2018**, *226*, 80–90, doi:10.1016/j.jamcollsurg.2017.10.010.

76. Mealer, M.; Jones, J.; Meek, P. Factors Affecting Resilience and Development of Posttraumatic Stress Disorder in Critical Care Nurses. *Am J Crit Care* **2017**, *26*, 184–192, doi:10.4037/ajcc2017798.

77. Shatté, A.; Perlman, A.; Smith, B.; Lynch, W.D. The Positive Effect of Resilience on Stress and Business Outcomes in Difficult Work Environments. *J Occup Environ Med* **2017**, *59*, 135–140, doi:10.1097/JOM.0000000000000914.

78. Saksvik-Lehouillier, I.; Bjorvatn, B.; Hetland, H.; Sandal, G.M.; Moen, B.E.; Magerøy, N.; Harvey, A.; Costa, G.; Pallesen, S. Personality Factors Predicting Changes in Shift Work Tolerance: A Longitudinal Study among Nurses Working Rotating Shifts. *Work & Stress* **2012**, *26*, 143–160, doi:10.1080/02678373.2012.686344.

79. Van Schrojenstein Lantman, M.; Mackus, M.; Otten, L.S.; de Kruijff, D.; van de Loo, A.J.; Kraneveld, A.D.; Garssen, J.; Verster, J.C. Mental Resilience, Perceived Immune Functioning, and Health. *J Multidiscip Healthc* **2017**, *10*, 107–112, doi:10.2147/JMDH.S130432.

80. Jo, I.; Lee, S.; Sung, G.; Kim, M.; Lee, S.; Park, J.; Lee, K. Relationship between Burnout and PTSD Symptoms in Firefighters: The Moderating Effects of a Sense of Calling to Firefighting. *Int Arch Occup Environ Health* **2018**, *91*, 117–123, doi:10.1007/s00420-017-1263-6.

81. Kim, W.; Bae, M.; Chang, S.-J.; Yoon, J.-H.; Jeong, D.Y.; Hyun, D.-S.; Ryu, H.-Y.; Park, K.-S.; Kim, M.-J.; Kim, C. Effect of Burnout on Post-Traumatic Stress Disorder Symptoms Among Firefighters in Korea: Data From the Firefighter Research on Enhancement of Safety & Health (FRESH). *J Prev Med Public Health* **2019**, *52*, 345–354, doi:10.3961/jpmph.19.116.

82. LaFauci Schutt, J.M.; Marotta, S.A. Personal and Environmental Predictors of Posttraumatic Stress in Emergency Management Professionals. *Psychological Trauma: Theory, Research, Practice, and Policy* **2011**, *3*, 8–15, doi:10.1037/a0020588.
