# Supplementary material for: Moderating effect of work fatigue on the association between resilience and posttraumatic stress symptoms: a cross-sectional multi-country study among pharmacists during the COVID-19 pandemic
Source: Biopsychosoc Med. 2024 Feb 19;18:4. doi: 10.1186/s13030-024-00300-0 (PMC10875825; doi:10.1186/s13030-024-00300-0)
Supplement: Supplementary file 2 — Additional file 2. [file 13030_2024_300_MOESM2_ESM.docx]

We would like to thank the reviewers for their time and efforts in reviewing our paper. Their comments definitely improved our manuscript. We hope the revised version is up to their expectations.

Reviewer #1: Thank you for submitting your research paper. This study was interesting because it investigated pharmacists' stress during the coronavirus pandemic and the psychological qualities needed to cope with it. This study showed that an individual's psychological qualities, such as resilience to cope with social stress, have a constant influence up to a certain stress load level. On the other hand, the authors note that the resilience do not work well in long-term, severe stress situations, such as the current coronavirus pandemic, and that maintaining an individual's physical and mental health requires social support. It also shows that it is essential. This is an especially important and reasonable result. I highly commend this study for demonstrating this.

However, before accepting this research into this journal, as a reviewer, I would like to request improvements in the following points.


major point

1 Problems with statistical methods
　
The authors state that they conducted a mediation analysis. However, it is still unclear how exactly this was implemented. If hierarchical multiple regression analysis is used, the description in Table 4 is insufficient. The hierarchy in which variables are added should be shown. Also, if a significant difference in the interaction term is shown, shouldn't a simple slope analysis be performed? Furthermore, when it is performed, a discussion based on the results should be added. Please check this point and make corrections as necessary.

We conducted a moderation analysis (term corrected in the statistical analysis paragraph; apologies for the typo mistake).

The PROCESS Macro divides the slopes into three slopes according to the mean, -1 SD and +1 SD of the moderator. We added the following sentence to the statistical analysis paragraph:

Interaction terms were probed by examining the association of the predictor with PTSD at the mean, 1 SD below the mean and 1 SD above the mean of the moderators (physical/mental/emotional work fatigue).

Next, regarding Table 5, the authors classify fatigue into three levels: Low, Moderate, and High, but the criteria are unclear. If the sample is divided into three groups based on the level of fatigue, a table should be created and the descriptive statistics should include the number of people in the three groups and the scores for Resilience, PTSD, etc. Furthermore, the statistics in Table 5 seem to show the influence of resilience on PTSD score, but the statistical method is unclear. Please check this point and make corrections if necessary.
We added the following sentence to the statistical analysis paragraph:

Interaction terms were probed by examining the association of the predictor with PTSD at the mean, 1 SD below the mean and 1 SD above the mean of the moderators (physical/mental/emotional work fatigue).

The whole analysis is done through the PROCESS Macro, where resilience was entered as the independent variable, each work fatigue subscale as a moderator in one model, and PTSD score as the dependent variable. We also entered all variables that showed a *p* < 0.25 in the bivariate analysis as covariates (confounding variables).

2 Minor point

Normally, the vertical lines on the table should not be drawn as much as possible. The visibility is poor so I hope they fix it.
We followed the journal’s instructions for the tables; however we removed the vertical lines as requested.

In the abstract and main text, only the P value of the regression analysis is listed, but since both β and p value are important, I would like this to be corrected.
Beta values were added to the abstract as requested.

I hope these points are carefully improved and your research paper is published.

Reviewer #2: Comments:
I would like to thank the authors for the opportunity to review this manuscript entitled "Moderating effect of work fatigue on the association between resilience and posttraumatic stress disorder: A cross-sectional multi-country study among pharmacists during the COVID-19 pandemic". They examined the moderating effect of work fatigue on the association between resilience and stress response. However, several limitations to this study and manuscript limit enthusiasm for this work. I note some of these considerations below:

#1:
The title of this study is "Moderating effect of work fatigue on the association between resilience and posttraumatic stress disorder: A cross-sectional multi country study among pharmacists during the COVID-19 pandemic." However, the title of this study is inappropriate because the study was not conducted on patients with PTSD.

Thank you for drawing our attention to this! We totally agree with you; we apologize for this mistake. “Posttraumatic stress disorder” was changed to “Posttraumatic stress symptoms”. Besides, this change has been made consistently throughout the paper.

#2:
The authors indicate that pharmacists may suffer PTSD due to COVID-19. However, I could not understand why they met the criteria A of the DSM. Please explain in detail.
And, even if they meet diagnostic criteria A for PTSD due to COVID-19, please describe whether the participants meet diagnostic criteria A for PTSD.

As we are investigating a sample of pharmacists and post-traumatic stress symptoms occurring on a continuum of severity, the part of the introduction related to DSM criteria has been considered as inappropriate and out of context. Therefore, it has been entirely removed from the paper. As mentioned above, the dependent variable here is “Posttraumatic stress symptoms” as assessed using the Impact of Event scale. The paper was consistently revised in accordance with your suggestions. We thank you for helping us improve the consistency and quality of our work.

The authors recruited the subjects from January to December 2021. I think the participants' condition varies depending on the time of the survey. Please add a reasonable explanation for the duration of the survey.
Yes, you are right; an explanation has been added as follows:

“The long period of data collection was due to the difficulty in availability of collaborators from the different countries involved, and in collecting data from pharmacists in the context of the COVID-19 situation, as this time of crisis substantially affected the daily practice of this population and required their priorities to be modified.”

In addition, we added this idea as a limitation:

“Subjects were recruited from January to December 2021; therefore, participants' conditions might have varied depending on the time of the survey.”

#3:
Please provide a rational explanation as to why the authors chose 8 countries as the target population for this study. Each country must have different cultures and policies on COVID-19.
Even though the authors surveyed 8 countries, the sample size is small (N =442) and the authors adjusted the countries when the analysis was performed.
What was the purpose of including 8 countries in this study?
And basically, it is recommended that an ethics application be filed for each country.

This research was designed as a multinational collaboration project. Several researchers from different institutions of different countries were invited to collaborate in our multinational project, and to join our team as co-investigators and co-authors. Only countries of researchers who agreed to collaborate were involved in this study. The protocol of the study was approved by the Ethics Committee of the home institution of the Principal Investigator (KI), which is an approach commonly adopted in multi-country research (as examples, please see 10.1007/s00127-022-02403-x; [10.1016/j.evolhumbehav.2022.08.003](https://doi.org/10.1016/j.evolhumbehav.2022.08.003)). All collaborators who collected data were asked to follow the ethical guidelines of their Institutional Review Board (IRB), acting either on the ethical approval of the Principal Investigator’s Ethical Approval or that received from their local IRBs.

#4:
The authors stated "Our samples consisted of pharmacists working in hospital or community pharmacy settings from eight countries and who were exposed to or worked with COVID-19 patients". However, In the Working in contact with COVID-19 patients section of Table 1, the authors indicated that there were 215 respondents who answered No. What does this mean?

The presence of an exposure to COVID-19 patients was not required as an inclusion criterion. We sincerely apologize for this wrong sentence. This was removed.

#5:
The authors stated "To calculate the sample size, we used G*Power software. The minimum required sample size was 389 participants, considering an alpha error of 5%, a power of 80%, a minimal model r-square of 5%, and allowing 15 predictors for inclusion in the model". What is this sample size for consideration?

The minimum sample to be required to have enough statistical power was 389. We ended up recruiting 442 pharmacists, which exceeds the minimum sample size. Therefore, our results have enough power to draw conclusions out of them.

#6:
The authors stated "The questionnaire was created using Google Forms, a cloud-based survey tool powered by Google™ and the questionnaire link was distributed online, using snowball sampling to enroll participants [49]".
Several studies have suggested that the quality of the survey may deteriorate in online surveys. Did the authors do anything to exclude those who answered inappropriately?

Unfortunately, we could not know which participants answered inappropriately (according to us, the answers were thorough and complete); however, we added your idea as a limitation:

The quality of the survey may not be high in online surveys; in addition, we could not know which participants answered the survey in an inappropriate way to exclude them.

Were there any rewards for participants? Also, I would like more detailed descriptions on how to recruit participants. These are very important.

We have already included this sentence in the first paragraph of the methods section:

Participants completed the survey voluntarily and **without remuneration**.

And, the authors stated "The online survey was formulated in English and translated to Arabic, Portuguese, Serbian and Polish according to the World Health Organization (WHO) translation guidelines. The research teams collaborated with a certified translator, consulted to detect discrepancies, solve them, validate and translate the questionnaire into the local language; this version was back-translated to English by a second certified translator in each country. A pilot test was done on 20 participants to make sure that the questions were well understood".
Did the authors obtain the rights to translate from the authors before translations, and did the authors examine the reliability and validity of the translations?
Permission to use the scales was obtained from respective authors. The reliability and validity of the translated scales was done by the research team in each country. More information can be added as supplementary material if deemed necessary.

#7:
The authors used the Depression Anxiety Stress Scale, but the results were not written.
This paragraph was removed.

#8:
The authors used the impact of event scale to assess the occurrence of PTSD. Did the participants reported traumatic stress event meet the DSM's criterion A? Have all exceeded the probable PTSD cutoff?

As explained above, post-traumatic stress symptoms occurring on a continuum of severity were assessed using the Impact of Event scale. No cutoff was used. The dependent variable was posttraumatic stress symptoms. Participants do not have to meet DSM criteria for PTSD. Any reference to PTSD or DSM was removed from the paper to keep consistency.

Reviewer #3: This study is a valuable work dealing with the mental health of pharmacists during a pandemic. However, a more careful description of the significance of this study's methodology is needed.

1) I wonder if the results could be affected by the prevalence of COVID-19 at the time of the responses to the survey. Also, were any of the targeted countries under lockdown at the time of the survey?

We added this idea to the limitations paragraph:

Subjects were recruited from January to December 2021; therefore, participants' conditions might have varied depending on the time of the survey.

2) Please describe for what purpose authors obtained data from multiple countries.

This choice was made to provide a broader overview of the relation between study variables in various countries and regions. Although the sample size was relatively small, we believe that controlling for country in our analyses has limited the country-level confounding effect. This explanation has been added to the revised text.

3) Please align the decimal places of the t-values in Table 5.

Done.
